# Development of a neonatal adverse event severity scale through a Delphi consensus approach

**DOI:** 10.1136/archdischild-2019-317399

**Published:** 2019-09-19

**Authors:** Thomas Salaets, Mark A Turner, Mary Short, Robert M Ward, Isamu Hokuto, Ronald L Ariagno, Agnes Klein, Sandra Beauman, Kelly Wade, Merran Thomson, Eve Roberts, Judy Harrison, Theresa Quinn, Gerri Baer, Jonathan Davis, Karel Allegaert

**Affiliations:** 1 Department of Development and Regeneration, KU Leuven, Leuven, Belgium; 2 Institute of Translational Medicine, University of Liverpool, Liverpool, UK; 3 Eli Lilly and Co, Indianapolis, Indiana, USA; 4 4Department of Pediatrics, Divisions of Neonatology and Clinical Pharmacology, University of Utah, Salt Lake City, Utah, USA; 5 Department of Pediatrics, St. Marianna University, Kawasaki, Japan; 6 Department Pediatrics-Neonatology, Stanford University School of Medicine, Palo Alto, California, USA; 7 Health Canada, Ottawa, Ontario, Canada; 8 Department of Pediatrics, University of New Mexico Health Sciences Center, Albuquerque, New Mexico, USA; 9 Division of Neonatology, Children's Hospital of Philadelphia, Philadelphia, Pennsylvania, USA; 10 Hillingdon Hospitals NHS Foundation Trust, Uxbridge, UK; 11 Institute of Translational Medicine, University of Liverpool, Liverpool, UK; 12 Maintenance and Support Services Organization, MedDRA, McLean, Virginia, USA; 13 Enterprise Vocabulary Services, National Cancer Institute, Bethesda, Maryland, USA; 14 Office of Pediatric Therapeutics, US Food and Drug Administration, Silver Spring, Maryland, USA; 15 Floating Hospital for Children at Tufts Medical Center, Boston, Massachusetts, USA; 16 Tufts Clinical and Translational Science Institute, Boston, Massachusetts, USA; 17 Department of Pediatrics, Division of Neonatology, Erasmus MC Sophia Kinderziekenhuis, Rotterdam, The Netherlands

**Keywords:** adverse event, severity grading, drug safety, neonate

## Abstract

**Background:**

Assessment of the seriousness, expectedness and causality are necessary for any adverse event (AE) in a clinical trial. In addition, assessing AE severity helps determine the importance of the AE in the clinical setting. Standardisation of AE severity criteria could make safety information more reliable and comparable across trials. Although standardised AE severity scales have been developed in other research fields, they are not suitable for use in neonates. The development of an AE severity scale to facilitate the conduct and interpretation of neonatal clinical trials is therefore urgently needed.

**Methods:**

A stepwise consensus process was undertaken within the International Neonatal Consortium (INC) with input from all relevant stakeholders. The consensus process included several rounds of surveys (based on a Delphi approach), face-to-face meetings and a pilot validation.

**Results:**

Neonatal AE severity was classified by five grades (mild, moderate, severe, life threatening or death). AE severity in neonates was defined by the effect of the AE on age appropriate behaviour, basal physiological functions and care changes in response to the AE. Pilot validation of the generic criteria revealed κ=0.23 and guided further refinement. This generic scale was applied to 35 typical and common neonatal AEs resulting in the INC neonatal AE severity scale (NAESS) V.1.0, which is now publicly available.

**Discussion:**

The INC NAESS is an ongoing effort that will be continuously updated. Future perspectives include further validation and the development of a training module for users.

**What is already known on this topic?**

- Communication of safety data between study investigators, sponsors and regulators remains suboptimal because of diverse ways of collection, reporting and assessment of adverse event information. In several research fields, severity scales have been developed to standardise adverse event severity reporting; however, the existing scales are not applicable to neonates.

**What this study adds?**

- This study describes a consensus process that led to the development of standard severity criteria for neonatal adverse events. The use of this tool could improve the quality of drug and device safety evaluations and facilitate the conduct of neonatal clinical trials.

## Introduction

An adverse event (AE) is defined as ‘any untoward medical occurrence associated with the use of a drug in humans, whether or not considered drug related’.^1–3^ In vulnerable populations such as critically ill neonates, background rates of mortality and morbidity are high.^4^ This leads to a higher incidence of reported AEs, not necessarily attributed to the investigational medicinal product (IMP) or device. However, when recognised and reported in a standardised manner, AEs can be important safety signals.

**Figure 1:**
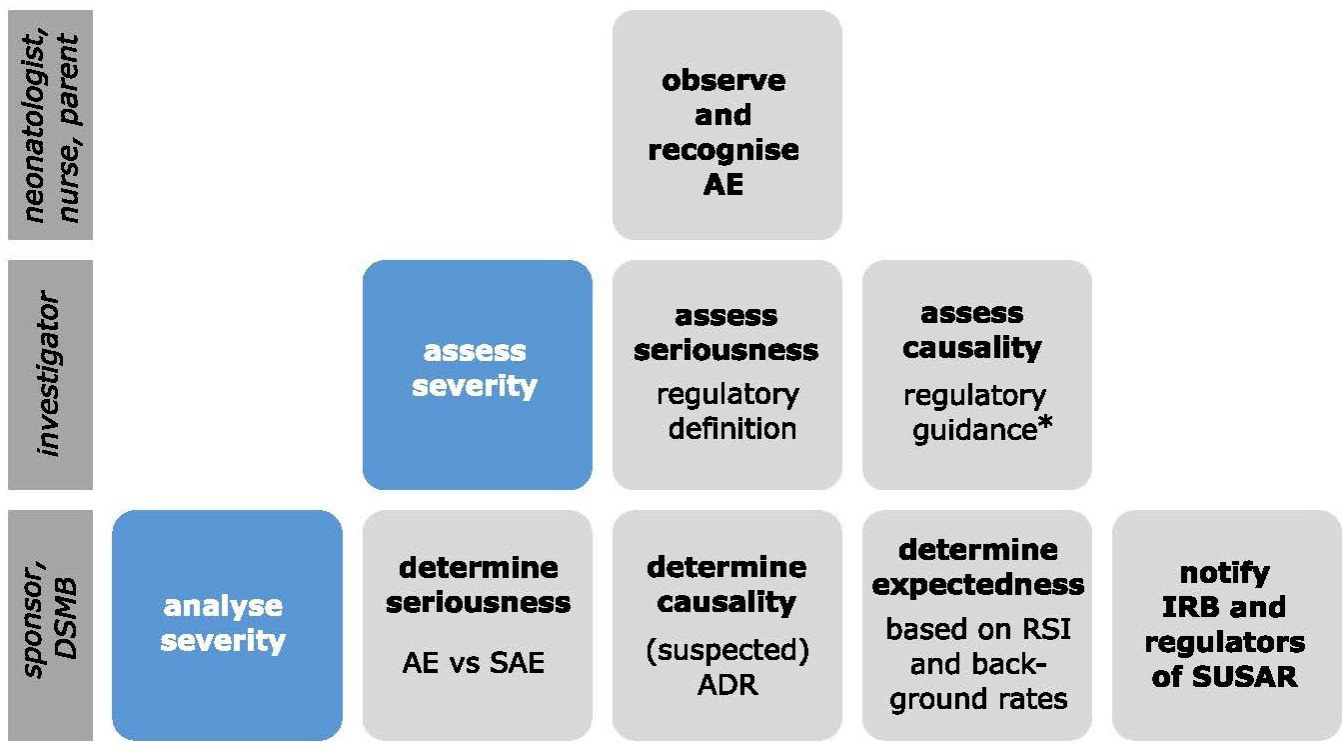
This figure summarises aspects of AEs that should be considered to account for safety reporting. It visualises responsibilities of the different actors and the currently available criteria and guidance. The figure is not intended to illustrate sequential activities. *Causality assessment relies on regulatory guidance; however, algorithms (eg, Du *et al* 6) have been developed for a neonatal population. AE, adverse event; DMSB, Data Safety Monitoring Boards; SAE, serious adverse event; ADR, adverse drug reaction; RSI, reference safety information; IRB, institutional review board; SUSAR, suspected unexpected serious adverse drug reaction.

Regulatory guidelines require investigators to assess whether an AE is serious and whether there is a reasonable possibility that it is related to IMP administration (causality) (figure 1).^1 5^ A strict regulatory definition exists for ‘seriousness’.^5^ Although causality involves clinical judgement, algorithms have been developed to make this assessment in a neonatal population more objective and homogeneous.^6 7^ Data Safety Monitoring Boards and sponsors review the investigator report of an AE and adjudicate whether the AE is expected based on known side effects (Reference Safety Information) and background complication rates.^5^ Finally, assessing the severity of an AE enhances the reporting process by capturing medical intensity.

Communication of safety data between study investigators, sponsors and regulators remains suboptimal because of diverse ways of collection, reporting and assessment. A common clinical research language, using standard terms and definitions, could facilitate responsible data sharing.^8 9^ Within neonatology, efforts have been directed towards developing standard terminology and definitions for AEs^10^ that integrate in to larger dictionaries such as the Thesaurus of the National Cancer Institute (NCI) or the Medical Dictionary for Regulatory Activities (MedDRA).^11 12^

Standardising criteria to report AE severity could make safety information more comparable across centres and trials and is a reasonable next step.^13^ In other research fields, toxicity tables and AE severity scales are commonly used.^14–16^ The most widely used example is the Common Terminology Criteria for Adverse Events (CTCAE), which was developed for oncology research.^17^ As the generic severity criteria of these scales are not applicable to neonates (table 1), we identified the need for a neonatal AE severity scale (NAESS) with criteria for specific neonatal AEs.

**Table 1:** Generic severity criteria of CTCAE, which are commonly used for adult and paediatric patients, but are not directly applicable to neonates

Grade 1	Grade 2	Grade 3	Grade 4	Grade 5
Mild	Moderate	Severe	Life threatening	Death
Mild; asymptomatic or mild symptoms; clinical or diagnostic observations only; intervention not indicated.	Moderate; minimal, local or non-invasive intervention indicated; limiting age-appropriate instrumental activities of daily living.	Severe or medically significant but not immediately life threatening; hospitalisation or prolongation of hospitalisation indicated; disabling; limiting self-care activities of daily living.	Life-threatening consequences; urgent intervention indicated.	Death related to AE.

## Methods

**Figure 2:**
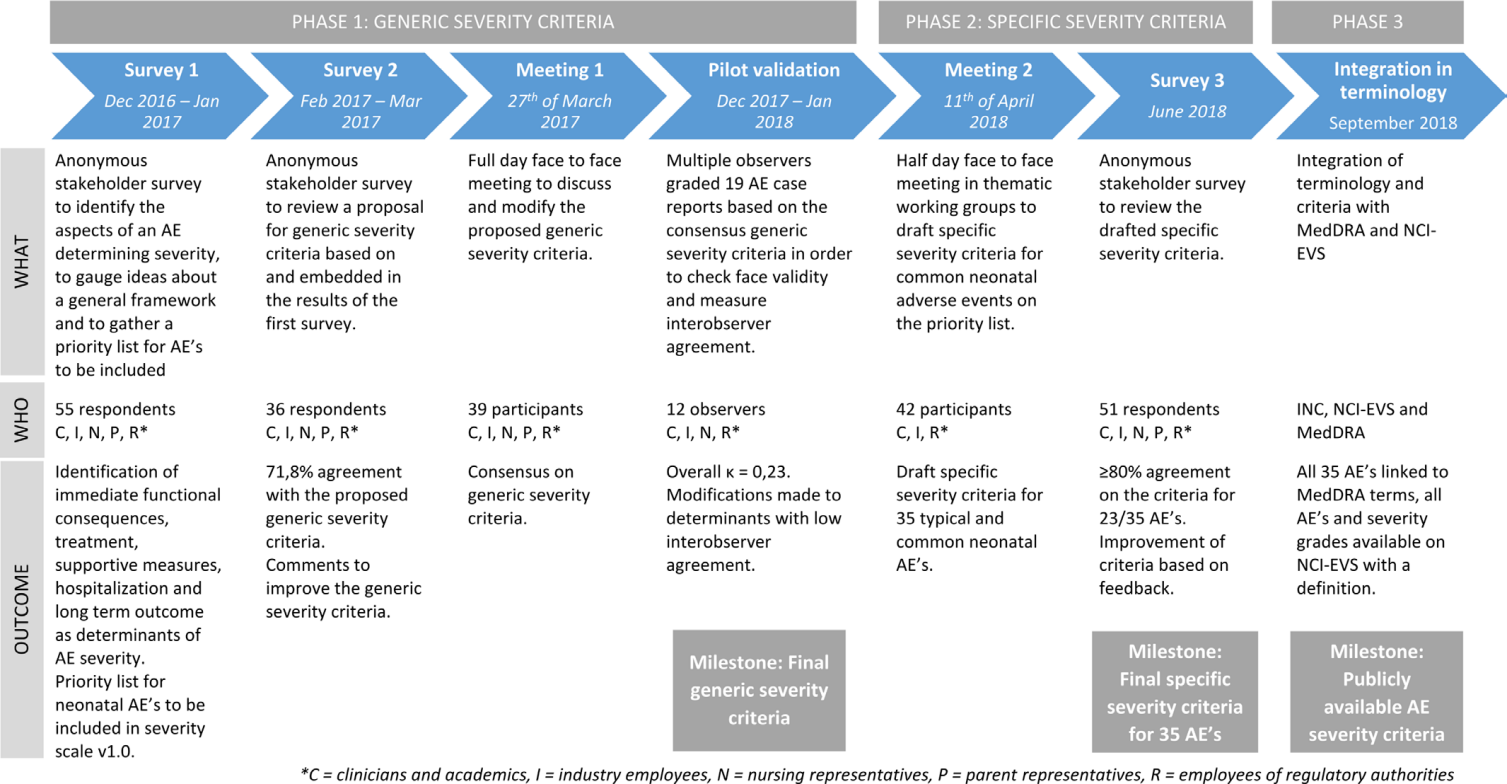
This figure gives an overview of the development process of the NAESS. Stakeholder involvement is indicated by C (clinicians), I (industry), N (nursing representatives), P (parent representatives) and R (regulatory authority employees). AE, adverse event; INC, International Neonatal Consortium; NCI-EVS, National Cancer Institute—Enterprise Vocabulary Services; MedDRA, Medical Dictionary for Regulatory Activities; NAESS, neonatal AE severity scale.

A stepwise consensus process, based on a Delphi approach,^18^ was undertaken between December 2016 and September 2018. First, generic severity criteria for neonatal AEs were developed. Next, these severity criteria were applied to frequently occurring neonatal AEs. Finally, the terminology was integrated into larger terminology dictionaries (figure 2). Full reports for every step can be found in the appendices.

### Stakeholder input

The NAESS was developed within the International Neonatal Consortium (INC). INC was established in 2015 in order to address regulatory and scientific challenges in the development of innovative drugs for neonates.^19^ Throughout the neonatal AE scale development, input was requested from multiple key stakeholders involved in neonatal drug development. Academic and non-academic clinicians and researchers, industry representatives, regulators, nursing and funding organisations and parent representatives from Canada, Europe, Japan and USA participated in the process (figure 2). Respondent and participant groups were expanded at every round to incorporate feedback from a maximal number of stakeholders.

### Development of generic severity criteria

Step 1: a first anonymous online survey was circulated through the network of INC and assessed in general terms regarding which aspects of AEs could be used as severity markers and provide input on the framework of NAESS.

Step 2: a second anonymous online survey presented the results and feedback of the first survey together with a proposal for generic severity criteria based on these results (Delphi approach).^18^

Step 3: in a face-to-face meeting, all components of the scale were discussed in the context of the feedback received in the surveys in order to achieve consensus on the generic severity criteria.

Step 4: pilot validation was undertaken to test the validity and interobserver agreement of the consensus generic severity criteria. Nineteen written case reports of AEs were provided by the University of Liverpool.^20^ All reports contained information on parameters, clinical and technical examinations, drug exposure and any resulting care changes. Twelve observers with different backgrounds from Canada, Europe, Japan and USA graded the severity of the 19 AEs using the proposed full generic severity scale and all individual markers (see online supplementary appendix 1). The results were analysed by calculating a free-marginal multirater kappa as a measure of interobserver reliability.^21 22^ The generic criteria were subsequently improved based on the results of this exercise.

10.1136/archdischild-2019-317399.supp1 Supplementary data

### Development of event specific severity criteria

Step 5: during a subsequent *face-to-face meeting*, thematic subgroups (neurological, cardiovascular, respiratory, gastrointestinal and infectious/general neonatology) drafted severity criteria for a list of specific neonatal AEs provided in step 1.

Step 6: the resulting specific severity criteria were evaluated in a final anonymous online survey. For all AEs with more than 20% disagreement, modifications were made in order to align all the key stakeholders. All modified criteria were approved in a final teleconference.

### Linking to existing terminology

Step 7: for each AE, a definition was used from the National Institute of Child Health and Human Development (NICHD) Pediatric AE Terminology if appropriate.^10^ Every AE was linked to the corresponding MedDRA Lowest Level Terms (LLTs). MedDRA Maintenance and Support Services Organization was contacted with a proposal to adjust or add terms if no suitable LLT was available. All criteria were added to the NCI Thesaurus.

## Results

In total, 109 members participated in the process leading to consensus on INC NAESS V.1.0. Participant numbers and background for all steps are summarised in figure 2 and online supplementary appendix 2. All participants who were involved in at least one step are listed in the Acknowledgement section.

10.1136/archdischild-2019-317399.supp2 Supplementary data

### Development of generic severity criteria

Step 1: e received 55 responses to the first survey. Immediate functional consequences (accepted by 81% of respondents), changes in treatment (82%), prolongation of hospitalisation (75%), supportive measures (85%) and long-term outcome (73%) were accepted as indicators of AE severity. Many comments referred to the feasibility of using long-term outcome to classify AE severity (see online supplementary appendix 3).

10.1136/archdischild-2019-317399.supp3 Supplementary data

Step 2: 36 respondents completed the second survey. Of the respondents, 72% agreed with the proposal for generic severity criteria based on immediate functional consequences, treatment (including supportive measures) and prolongation of the initial hospitalisation. The remaining 28% of respondents suggested adjustments (see online supplementary appendix 4).

10.1136/archdischild-2019-317399.supp4 Supplementary data

Step 3: 39 experts participated in the first face-to-face meeting trying to define generic severity criteria for neonates. Consistent with other AE severity scales, severity was subdivided into five categories: mild, moderate, severe, life threatening and death. Immediate functional consequences (on age appropriate behaviour and basal physiological functions), together with resulting care changes were established as the parameters of the generic AE severity scale. Additionally, the stakeholder group agreed that this AE severity scale would pertain to neonates <44 weeks postmenstrual age.^23 24^

Step 4: the proposed consensus scale resulted in a multirater free-marginal κ of 0.23 (ranging from −0.03 to 0.59 for individual AEs). Severity evaluations based on ‘care changes’ alone resulted in suboptimal agreement. Furthermore, marked intraobserver and interobserver variability was noted in how the 12 observers weighted the different factors involved in the determination for the final severity grade (see online supplementary appendix 1).

Examples of minor and major care changes were added for clarification. It was also specified that if individual determinants of severity resulted in a discrepant severity grade, the highest grade would be reported. These changes resulted in the final generic severity criteria as shown in table 2.

**Table 2:** Generic severity criteria of INC NAESS developed for use in neonates

Grade 1	Grade 2	Grade 3	Grade 4	Grade 5
Mild	Moderate	Severe	Life threatening	Death
Mild; asymptomatic or mild symptoms; clinical or diagnostic observations only; no change in baseline age-appropriate behaviour*; no change in baseline care or monitoring indicated.	Moderate; resulting in minor changes of baseline age-appropriate behaviour*; requiring minor changes in baseline care or monitoring.*†	Severe; resulting in major changes of baseline age-appropriate behaviour* or non-life-threatening changes in basal physiological processes†; requiring major change in baseline care or monitoring.*‡	Life threatening; *resulting in life-threatening changes in basal physiological processes†; requiring urgent major change in baseline care.*	Death related to AE.

If the different factors of this scale result in conflicting severity grades, the highest grade should be reported. *Italics* indicate the differences with the adult generic severity criteria of CTCAE.

*Age-appropriate behaviour refers to oral feeding behaviour, voluntary movements and activity, crying pattern, social interactions and perception of pain.

†Basal physiological processes refer to oxygenation, ventilation, tissue perfusion, metabolic stability and organ functioning.

‡Minor care changes constitute: brief, local, non-invasive or symptomatic treatments.

§Major care changes constitute: surgery, addition of long-term treatment, upscaling care level.

CTCAE, Common Terminology Criteria for Adverse Events.

### Development of event specific severity criteria

Step 5: 42 professionals, in five thematic subgroups, drafted the specific severity criteria for the 35 common neonatal AEs (table 3) during a second face-to-face meeting. Specific disease markers were used as severity criteria if it was determined that they reflected the factors incorporated in the generic severity scale. AEs based on laboratory values were not included in this first version because reliable reference values for normality were not available (for postmenstrual and postnatal age).

**Table 3:** AEs included in the current version of INC NAESS

AEs in INC neonatal AE severity scale	
**Neurological**	**Respiratory**
Neonatal convulsion	Infantile apnoea
Neonatal epileptic seizure	Neonatal respiratory insufficiency
Neonatal intraventricular haemorrhage*	Neonatal respiratory distress syndrome
Retinopathy of prematurity*	Neonatal pulmonary haemorrhage*
Hypoxic ischaemic encephalopathy	Persistent pulmonary hypertension of the newborn*
Periventricular leukomalacia*	Neonatal pneumothorax*
Infant irritability	Bronchopulmonary dysplasia
Infant sedation*	**Gastrointestinal**
**Cardiovascular**	Necrotising enterocolitis
Neonatal hypotension	Neonatal diarrhoea*
Neonatal hypertension*	Infantile vomiting*
Neonatal sinus tachycardia*	Feeding intolerance
Neonatal sinus bradycardia	Neonatal gastrointestinal bleeding*
Neonatal tachyarrhythmia*	Neonatal spontaneous intestinal perforation*
Neonatal bradycardia	Neonatal constipation*
Neonatal oedema*	**General**
Neonatal coagulation disorder*	Neonatal rash*
**Infectious**	Neonatal administration site complication*
Neonatal culture positive sepsis*	Neonatal fever*
Neonatal culture negative sepsis*	

For these 35 AEs, specific severity criteria were defined.

AE, adverse event; INC, International Neonatal Consortium; NAESS, neonatal AE severity scale.

Step 6: among the 51 respondents of the final survey, there was ≥80% agreement for 23 out of 35 of the draft criteria (indicated with an asterisk*). For the remaining 12 AEs, criteria were then adjusted (see online supplementary appendix 5) and approved in a final teleconference. The resulting specific severity criteria for all selected 35 AEs can be found in the INC AE severity scale V.1.0 (online supplementary appendix 6); as an example, the specific severity criteria for neonatal convulsion are shown in table 4.

10.1136/archdischild-2019-317399.supp5 Supplementary data

10.1136/archdischild-2019-317399.supp6 Supplementary data

**Table 4:** Severity criteria for neonatal convulsions, as an example of the specific severity criteria per AE given in INC NAESS (online supplementary appendix 6)

Grade 1	Grade 2	Grade 3	Grade 4	Grade 5
Mild	Moderate	Severe	Life threatening	Death
**Neonatal convulsion** Definition C154952 │ 10028932: *Sudden, involuntary, rapid, rhythmic or stereotyped skeletal muscular contractions in a newborn*.				
Single, self-limited suspected seizure, no treatment.	Suspected seizures controlled with one anti-seizure drug (no recurrence within 3 days after treatment).	Suspected seizures uncontrolled with one antiseizure drug (recurrence within 3 days after treatment or requiring two or more antiseizure drugs).	Suspected seizures with life-threatening consequences (eg, need for ventilation); suspected status epilepticus* despite multiple anti-seizure drugs.	Death related to suspected seizures.

*>30 min duration of convulsions within any 60 min period.

AE, adverse event; INC, International Neonatal Consortium; NAESS, neonatal AE severity scale.

### Linking to existing terminology

For 23 (66%) of the AEs in V.1.0 definitions were taken directly from the NICHD Paediatric AE Terminology. Nineteen (54%) of the AEs could be linked to an existing LLT in MedDRA V.21.0. New LLTs were added in MedDRA V.22.0 to match the remaining AEs. For all AEs and severity grades, specific codes for the NCI Thesaurus were generated. The terminology was made publicly available on the NCI Thesaurus (https://evs.nci.nih.gov/ftp1/INC/Adverse_Events_Terminology/).

## Discussion

This paper describes the development of the INC NAESS. It provides criteria that guide investigators and clinicians in assessing severity of AEs. It was developed to increase the quality of safety information.

### Generic severity criteria

The common framework of the AE severity scale is contained in generic criteria shown in table 2. It defines suitable severity markers for neonates, in contrast to criteria used in other populations (eg, CTCAE; table 1). First, impact on age-appropriate activities is included, describing the signs exhibited by the neonate (feeding behaviour, voluntary movements, activity, crying, social interactions and signs of pain). The impact on basal physiological processes (changes in oxygenation, ventilation, circulation, metabolic stability and organ function) is a second severity marker. A third severity marker is the change in care in response to the AE, as it reflects the severity of the underlying event and is indicative of additional stress. All of this information is readily available after the AE occurs and permits immediate severity grading.

It is important to note that for all determinants, only changes from the baseline condition due to the AE should be considered. Also, even though these criteria provide guidance, some form of clinical judgement (and thus subjectivity) remains inherent in the severity assessment.

The final decision was not to include long-term outcome as a marker of AE severity, as it might be difficult to establish a direct causal link. Furthermore, the overall goal of this scale is to create reliable and immediate safety signals prompting awareness, which is not compatible with assessing the severity of an event when the final outcome is only known years later. Despite this decision, examining the long-term outcome associated with a neonatal drug exposure remains a crucial effort that should be encouraged.^24^

### Validation

The goal of a standardised severity scale is to reduce subjectivity in severity assessments and thus reduce interobserver variability. Our pilot validation exercise on the consensus generic severity criteria revealed only fair agreement (κ=0.23) among observers of different backgrounds. This seems less rigorous than what is published for other severity scales (eg, CTCAE^25^ and SAVES-2 (Spinal Adverse Event Severity System, version 2)).^26^ It should be noted that our results were obtained by applying a generic scale, which is purposely broad and thus provides less direct guidance. Furthermore, our pilot validation was performed as a part of an optimisation process, resulting in improved final generic severity criteria. Future plans include measurement of interobserver agreement with the final scale on prospectively collected data in order to show the benefit of using standardised criteria for the severity of neonatal AEs.

### INC NAESS V.1.0

The current version (V.1.0) contains specific severity criteria for 35 routinely encountered neonatal AEs and more will be added. For instance, laboratory-based AEs will be included when age-appropriate reference values become available. The severity of AEs currently not included in INC NAESS V.1.0 can be estimated by applying the generic criteria.

This INC NAESS V.1.0 is now publicly available in the NCI Thesaurus. This platform allows end users to embed the criteria relevant to a particular project within the concept information. Its presence in the Thesaurus improves dissemination to a relevant audience. Finally, NCI Thesaurus users can request the addition of new AEs or modifications of existing criteria that permits the terminology to be revised as needed. This ensures the sustainability of the INC NAESS.

The INC NAESS is a continuous work in progress. We are preparing to expand the number of AEs included and encourage readers to suggest new AEs for addition or comment on existing criteria if deemed not appropriate through the NCI Thesaurus website.

### Anticipated benefits

Severity assessments provide a nuanced clinical appreciation of an AE. Standardising this information leads to more reliable and comparable information that can facilitate regulatory safety evaluations of drugs. Furthermore, it can improve scientific communication on AEs in publications. The availability of a standardised severity scale can also facilitate the conduct of clinical trials in neonates. For example, dose reductions can be recommended in response to severe AEs. In the neonatal population where the background rates of clinical AEs are high, protocols could stratify reporting obligations for different severity grades and background rates of AEs. This could enhance consistent AE reporting and reduce the administrative burden associated with neonatal research.

Finally, the scale could also be used in routine clinical care and for postmarketing pharmacovigilance. Neonates are vulnerable to adverse drug reactions (ADR) in clinical care, but these events are under-reported.^27^ NAESS was developed to classify AEs, which would complement neonatal algorithms used to identify ADRs.^6 7^ A standardised evaluation of severity would add information that is useful for pharmacovigilance, quality improvement projects, observational studies or registries and can help establish risk/benefit ratios of common therapies.^28^

## Conclusion

In conclusion, a NAESS containing both generic and specific criteria for 35 common neonatal AEs was developed in collaboration with key stakeholders involved in neonatal drug development. The use of this tool can improve the quality of drug and device safety evaluations and can facilitate the conduct of neonatal clinical trials. Future perspectives include validation research to assess interobserver agreement and the addition of more AEs.
