# Supplementary material for: Development of a neonatal adverse event severity scale through a Delphi consensus approach
Source: Arch Dis Child. 2019 Sep 19;104(12):1167–73. doi: 10.1136/archdischild-2019-317399 (PMC6943241; doi:10.1136/archdischild-2019-317399)
Supplement: Supplementary data [file archdischild-2019-317399supp001.pdf]

Appendix 1

Pilot validation study

Objective

Based on a modified Delphi-process and a face-to-face meeting, generic severity criteria were proposed in March 2017 (supplementary table 1). A pilot validation was consequently undertaken in order to validate the proposed severity criteria. The goal was to: 1) check face validity of the criteria on real life cases, 2) evaluate interobserver agreement of the scale and 3) evaluate the interobserver agreement on the different individual severity determinants in the scale to assess their performance and eventually improve the criteria.

Grade 1	Grade 2	Grade 3	Grade 4	Grade 5
Mild	Moderate	Severe	Life threatening	Death
Mild; asymptomatic or mild symptoms; clinical or diagnostic observations only; no change in baseline age-appropriate behavior*, no change in baseline care or monitoring indicated	Moderate; resulting in minor changes of baseline age-appropriate behavior*, requiring minor changes in baseline care or monitoring	Severe; resulting in major changes of baseline age-appropriate behavior* and/or non-life threatening changes in basal physiological processes**, requiring major change in baseline care or monitoring	Life-threatening; Resulting in life-threatening changes in basal physiological processes**	Death related to AE
*Age-appropriate behavior refers to oral feeding behavior, voluntary movements and activity, crying pattern, social interactions and perception of pain.<br>**Basal physiological processes refer to oxygenation, ventilation, tissue perfusion, metabolic stability and organ functioning.				

Supplementary table 1: Proposed generic severity criteria – March 2017

Methods

Nineteen written case reports of adverse events occurring in clinical practice in a neonatology ward were provided by the University of Liverpool (5). All reports contained a description of the event, with all relevant information on parameters, clinical status, technical examinations, drug exposure and care changes. Twelve observers experienced in clinical research in neonatology graded the severity of the adverse events. Among the twelve observers were 7 clinicians, 2 industry representatives, 2 employees of regulatory authorities and 1 nursing representative. They assigned an overall severity grade to each adverse event (1: mild, 2: moderate, 3: severe, 4: life-threatening or 5: death) based on the generic severity criteria visualized in supplementary table 1. Furthermore the observers were also asked to grade severity based on all individual severity determinants (consequences on age appropriate behavior, consequences on basal physiological processes and care changes). Finally the observers could give free field comments. The results were analyzed by calculating a free-marginal multi-rater kappa, as a measure for interobserver agreement (4). Kappa values were interpreted according to the guidance from Altman (2): poor <0.2; fair 0.21–0.40; moderate 0.41–0.60; good 0.61–0.80; and very good 0.81–1.00 agreement.

Results

For the overall scale a κ of 0.23 was obtained, ranging from -0.03 to 0.59 for individual adverse events. There was no clear trend in better or worse agreement depending on the background of the observers (supplementary table 2). The κ values for consequences on age appropriate behavior, for consequences on basal physiological processes and for care changes individually were 0.85; 0.34 and 0.29 respectively. Looking at the individual subentities of changes in basal physiological processes (oxygenation, ventilation, perfusion, metabolic stability and organ functioning) κ values of 0.51 to 0.95 were obtained (supplementary table 3).

There was a marked intra- and interobserver variability in how observers combined the different factors in to a final severity grade.

	Clinician 1	Clinician 2	Clinician 3	Clinician 4	Clinician 5	Clinician 6	Clinician 7	Nurse 1	Regulator 1	Regulator 2	Industry 1	Industry 2
Clinician 1		-0.12	0.51	-0.12	0.30	0.30	0.02	0.09	0.65	0.30	0.09	0.30
Clinician 2			-0.05	0.37	-0.05	0.16	0.37	0.30	-0.05	0.09	0.16	0.23
Clinician 3				0.09	0.30	0.37	-0.12	0.23	0.30	0.44	0.37	0.44
Clinician 4					0.16	0.23	0.58	0.58	-0.19	0.02	0.23	0.30
Clinician 5						0.30	0.16	0.30	0.51	0.16	0.58	0.23
Clinician 6							0.02	0.23	0.23	0.23	0.44	0.30
Clinician 7								0.44	0.09	0.09	0.16	0.23
Nurse 1									0.16	0.23	0.44	0.37
Regulator 1										0.23	0.30	0.02
Regulator 2											0.30	0.16
Industry 1												0.51
Industry 2												

Supplementary table 2: Individual interobserver agreement between observers (pairwise Cohen's kappa)

Determinant	Free-marginal multi-rater kappa
Age appropriate behavior	0.89
Basal physiological processes	0.34
Oxygenation	0.51
Ventilation	0.61
Perfusion	0.95
Metabolic stability	0.86
Organ functioning	0.67
Care changes	0.29

Supplementary table 3: Interobserver agreement for severity grading based on individual determinants of the severity scale (free-marginal multi-rater kappa)

Discussion

This pilot validation exercise revealed fair agreement (κ = 0.23) of observers of different backgrounds using the proposed adverse event severity scale. However the analysis is not directly comparable, there seems to be slightly less agreement then for other adverse event severity scales in other populations. Two clinicians rating the severity of constipation, diarrhea, dyspnea, fatigue, nausea, neuropathy and vomiting of oncology trial participants using the CTCAE severity scale, obtained intraclass correlation coefficients of 0.46 to 0.71 (1). Multiple observers rating case reports of adverse events after spinal surgery with a purpose developed severity table (SAVES-2) resulted in an intraclass correlation coefficient of 0.75 (3). If an intraclass correlation coefficient (agreement measure for continuous variables) was calculated for overall severity in our sample this resulted in a value of 0.39. This thus results in a slightly more optimistic value than the Fleiss' κ of 0.23 which we used for analysis (and which is an agreement measure for categorical variables).

We identified two main reasons for the suboptimal performance of the proposed severity scale. First, this scale consists of generic criteria that are kept very wide on purpose in order to be applicable on any given adverse event. This however makes them less guiding, and leaves more space for interpretation. Assessing the interobserver agreement of the final specific severity criteria would intuitively make the results more comparable to the mentioned literature (1, 3).

Second, the results of the individual determinant analysis indicate lowest interobserver agreement for assessing care changes, and a wide variability in how observers combine the different factors in to a final

severity grade. Hence, some modifications and clarifications in the generic severity scale were made in order to reduce variability in assessing these suboptimal components. The final scale is visualized in supplementary table 4. Further, also the need for a brief and simple training tool for observers was identified.

Grade 1	Grade 2	Grade 3	Grade 4	Grade 5
Mild	Moderate	Severe	Life threatening	Death
Mild; asymptomatic or mild symptoms; clinical or diagnostic observations only; no change in baseline age-appropriate behavior*; no change in baseline care or monitoring indicated	Moderate; resulting in minor changes of baseline age-appropriate behavior*; requiring minor changes in baseline care or monitoring***	Severe; resulting in major changes of baseline age-appropriate behavior* or non-life threatening changes in basal physiological processes**; requiring major change in baseline care or monitoring****	Life-threatening; Resulting in life-threatening changes in basal physiological processes**; requiring urgent major change in baseline care	Death related to AE
*Age-appropriate behavior refers to oral feeding behavior, voluntary movements and activity, crying pattern, social interactions and perception of pain.<br>**Basal physiological processes refer to oxygenation, ventilation, tissue perfusion, metabolic stability and organ functioning.<br>***Minor care changes constitute: brief, local, non-invasive or symptomatic treatments<br>***Major care changes constitute: surgery, addition of long term treatment, upscaling care level				
If the different factors of this scale result in conflicting severity grades, **the highest grade** should be reported.				

Supplementary table 4: Final generic severity criteria with modifications after pilot validation – March 2018

References:

1. **Atkinson TM, Li Y, Coffey CW, Sit L, Shaw M, Lavene D, Bennett AV, Fruscione M, Rogak L, Hay J, Gönen M, Schrag D, and Basch E**. Reliability of adverse symptom event reporting by clinicians. *Qual Life Res* 21: 1159-1164, 2012.
2. **Altman DG**. *Practical statistics for medical research*. London: Chapman & Hall, 1991.
3. **Rampersaud YR, Anderson PA, Dimar JR, 2nd, and Fisher CG**. Spinal Adverse Events Severity System, version 2 (SAVES-V2): inter- and intraobserver reliability assessment. *J Neurosurg Spine* 25: 256-263, 2016.
4. **Randolph JJ**. Free-marginal multirater kappa: An alternative to Fleiss´ fixed-marginal multirater kappa. In: *Joensuu University Learning and Instruction Symposium*. Joensuu, Finland: 2005.
5. **Roberts E, Hawcutt D, and Turner M**. Adrin 1 methodology study: adverse drug reactions in neonates: what are the best ways to evaluate suspected adverse drug reactions in neonates? *Archives of Disease in Childhood* 102: A11.12-A11, 2017.
