# Supplementary material for: Development of a neonatal adverse event severity scale through a Delphi consensus approach
Source: Arch Dis Child. 2019 Sep 19;104(12):1167–73. doi: 10.1136/archdischild-2019-317399 (PMC6943241; doi:10.1136/archdischild-2019-317399)
Supplement: Supplementary data [file archdischild-2019-317399supp002.pdf]

Appendix 2

Background of participants to all steps of consensus process

Survey 1 (55 respondents)

Academia	31 (56%)
Industry	10 (18%)
Regulatory authority	3 (5%)
Nursing organization	4 (7%)
Parent organization	1 (2%)
Other: government, health care organization, public hospital, …	6 (11%)

Survey 2 (36 respondents)

Academia	18 (50%)
Industry	7 (19%)
Regulatory authority	1 (3%)
Nursing organization	6 (17%)
Parent organization	1 (3%)
Other: government, health care organization, public hospital, …	3 (8%)

Face to face meeting 1 (39 participants)

Academia	19 (49%)
Industry	7 (18%)
Regulatory authority	8 (21%)
Nursing organization	1 (3%)
Parent organization	1 (3%)
Other: government, health care organization, public hospital, …	3 (8%)

Pilot validation (12 participants)

Academia	7 (58%)
Industry	2 (17%)
Regulatory authority	2 (17%)

Nursing organization	1 (8%)

Face to face meeting 2 (42 participants)

Academia	18 (43%)
Industry	15 (36%)
Regulatory authority	8 (19%)
Other: government, health care organization, public hospital, …	1 (2%)

Survey 3 (51 participants)

Academia	24 (47%)
Industry	14 (27%)
Regulatory authority	6 (12%)
Nursing organization	4 (8%)
Parent organization	1 (2%)
Other: government, health care organization, public hospital, …	2 (4%)
