# Supplementary material for: Development of a neonatal adverse event severity scale through a Delphi consensus approach
Source: Arch Dis Child. 2019 Sep 19;104(12):1167–73. doi: 10.1136/archdischild-2019-317399 (PMC6943241; doi:10.1136/archdischild-2019-317399)
Supplement: Supplementary data [file archdischild-2019-317399supp003.pdf]

Appendix 3

Report of survey 1

Introduction

This report presents the results of the first questionnaire of the (modified) Delphi process that has been undertaken within INC (International Neonatal Consortium) on severity grading of neonatal adverse events (AE's). This first questionnaire explored the ideas of relevant stakeholders on generic criteria for severity of neonatal AE's. This document shows the results of the 15 questions (in blue boxes) together with the background that has been provided to the respondents (in the same order as presented to the respondents).

Originally this effort was part of the Everrest project (a multicenter phase I/IIa trial to assess the safety and efficacy of vascular endothelial growth factor (VEGF) gene therapy as a treatment for severe early onset fetal growth restriction. [1] A similar exercise is currently ongoing for maternal and fetal adverse events. The effort has been endorsed by INC, in order to find a widely supported consensus among stakeholders in neonatal clinical trials.

Methods

For this first questionnaire, feedback was received from 55 relevant **stakeholders**. Due to problems with the identifiers in the questionnaire we did not identify all respondents, however we know that we achieved a broad panel with different backgrounds.

Academia	31 (56%)
Industry	10 (18%)
Regulatory authority	3 (5%)
Nursing organization	4 (7%)
Parent organization	1 (2%)
Other: government, health care organization, public hospital, …	6 (11%)

Table 1: Affiliations of respondents for questionnaire 1

Standardization and severity grading of neonatal adverse events: the current status

Defining adverse events

Before considering criteria of severity, we have to agree on definitions of neonatal adverse events. In an ideal scenario, these definitions are linked to internationally accepted definition registers.

The Medical Dictionary for Regulatory Activities (MedDRA) was developed by the International Conference on Harmonization of Technical Requirements for Pharmaceuticals for Human Use (ICH) to provide standard terms for adverse events, and is widely used by clinical researchers, regulatory authorities, and pharmaceutical companies. These terms are used for coding adverse events which occur in clinical trials or during post-market safety monitoring. They are available in several languages. In order to make the adverse event grading criteria as widely applicable as possible, it seems sensible to use the MedDRA preferred terms where possible. If the MedDRA terms are not considered adequate, it is possible to request that terms be added to the list.

Alternative systems include the International Statistical Classification of Diseases and Related Health Problems (ICD) and the WHO Adverse Reaction Terminology (Table 1).

Name		Purpose	Terms	Official Languages
Medical Dictionary for Regulatory Activities (MedDRA)		International safety monitoring of medicinal products	Over 70,000	11
International Statistical Classification of Diseases and Related Health Problems (ICD)	Internationally	International monitoring of diseases, signs, and symptoms	Over 16,000	6
	United States	Healthcare and insurance administration	Over 76,000	
WHO Adverse Reaction Terminology		International safety monitoring of medicinal products	2,000	6

Table 2: Comparison of three 'standard term' systems

In early 2009, the National Institute of Child Health and Human Development (NICHD, USA) initiated an ongoing effort to establish a core library of harmonized pediatric terms, beginning with a focus on neonatal and infant examination concepts as part of a broader terminology framework. [3] All terminology is added to NCI Thesaurus (NCIt) and as a list accessible through Enterprise Vocabulary Services (EVS). [4] Within this framework a list of Pediatric Adverse Events Terminology was published in 2014, which is mapped to MedDRA. This list also includes neonatal adverse events, some examples are shown in table 2.

Reference	Term	Definition
C84915	Necrotizing Enterocolitis	A fulminating disease of neonates in which there is extensive mucosal ulceration, pseudomembrane formation, submucosal hemorrhage, and necrosis usually of the right colon, cecum, terminal ileum, and appendix, possibly due to perinatal intestinal ischemia and bacterial invasion. Progression can lead to necrosis, perforation and/or scarring of the intestinal tract.
C87101	Neonatal Abstinence Syndrome	A constellation of neurobehavioral features observed in a neonate following antenatal exposure to drugs including opioids, benzodiazepines, and selective serotonin reuptake inhibitors.
C90599	Bronchopulmonary Dysplasia	Chronic lung disease requiring treatment with oxygen for at least 28 days and with a spectrum of severity from mild to severe, that predominantly affects premature infants. While the radiologic pattern is typical in the closer to term patient, the pattern in the small preterm infant is very non-discrete and variable.
C99013	Periventricular Leukomalacia	Necrosis and softening of the white matter around the ventricles in the brain. It is usually seen in preterm infants and caused by lack of blood flow and oxygenation in the periventricular brain parenchyma.
Examples of severity that may need revision		
C99057	Severe Bronchopulmonary Dysplasia	Bronchopulmonary dysplasia that results in severe respiratory complications. The infants with severe symptoms are usually premature, have low birth weight, and the oxygen and ventilation support requirements are increased.
C99138	Germinal Matrix Hemorrhage of Newborn	Bleeding into the thick layer of immature cells under the ependymal lining at the ventrolateral aspect of the lateral cerebral ventricles of a newborn. (Grade 1 IVH)

Salaets T, *et al. Arch Dis Child* 2019; 104:1167–1173. doi: 10.1136/archdischild-2019-317399

C99139	Intraventricular Hemorrhage of the Newborn without Ventricular Dilatation	Bleeding into the lateral cerebral ventricles of a newborn infant without acute ventricular dilatation. (Grade 2 UVH)
C99140	Intraventricular Hemorrhage of the Newborn with Ventricular Dilatation	Bleeding into the lateral cerebral ventricles of a newborn infant with ventricular dilatation directly attributable to the acute bleeding. (Grade 3 IVH)

Table 3: Definitions of selected conditions that we propose to use and severity assessments that may need revision.

These definitions are the result of considerable effort by a large number of people including representatives of the NICHD, the NCI EVS, and three additional neonatal research networks: Vermont-Oxford Network (VON), Children's Hospital Neonatal Database (CHND), the Neonatal Research Network (NRN) to support the reporting of neonatal events in research and the clinical setting.

Q1: We will use the definitions from the NICHD Pediatric Adverse Events Terminology, which were mapped to MedDRA previously, as a pragmatic starting point for adverse event definitions in this future consensus document. Do you have any objections to this?

Answers: 55

96% (53) of respondents agreed to use the NICHD Pediatric Adverse Events Terminology. Only two respondents objected. Two respondents commented that many of the definitions are pathology or etiology based, and this is often hard to use in a clinical research setting. One respondent commented that some definitions (BPD) are outdated.

Evaluating severity of adverse events

An adverse event is “any untoward medical occurrence in a patient or clinical trial participant administered a medicinal product and which does not necessarily have a causal relationship with this product”. When an adverse event occurs it must be recorded in the medical records and can be reported to the sponsor and competent authority as described in the protocol. As part of this process it must be decided whether (1) the event meets the European Medicines Agency (EMA) / Food and Drug Administration (FDA) definition of ‘serious’ and whether (2) it is related to the administration of the medicinal product (Figure 1). This determines whether it is an adverse event (AE), serious adverse event (SAE), adverse reaction (AR), or serious adverse reaction (SAR).

The above are regulatory obligations, but more detailed safety information could and should be obtained, for example on a 5 point scale to assess clinical severity. Ideally this would be done using standard definitions and severity grading criteria, which we aim to develop in this process. This grading could contribute to the comparison of benefit-risk balance between two or more arms of an interventional trial. Additionally, common definitions would help compare effects of interventions across trials

Salaets T, *et al. Arch Dis Child* 2019; 104:1167–1173. doi: 10.1136/archdischild-2019-317399

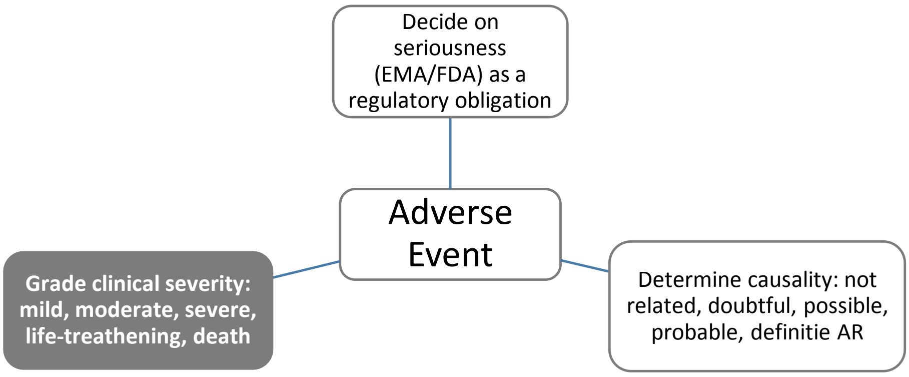

Figure 1: Issues to be considered when evaluating an adverse event

Serious Adverse Events

A Serious Adverse Event is defined by the International Conference on Harmonization Tripartite Guideline [5](encompassing the regulatory authorities of Europe, Japan, and the United States of America) as any untoward medical occurrence that:

- results in death
- is life-threatening (The term "life-threatening" in the definition of "serious" refers to an event in which the patient was at risk of death at the time of the event; it does not refer to an event which hypothetically might have caused death if it were more severe).
- requires inpatient hospitalization or prolongation of existing hospitalization,
- results in persistent or significant disability/incapacity, or
- is a congenital anomaly/birth defect.

"Seriousness" includes severity-information, but it is emphasized in the guideline that "serious" and "severe" are not synonymous. "Seriousness" is used to define regulatory reporting obligations, however it is felt that this dichotomous distinction is insufficient to give sufficient severity information.

Severity grading

Severity is a separate concept that is generally recorded in 5 grades:

Grade 1	Grade 2	Grade 3	Grade 4	Grade 5
Mild	Moderate	Severe	Life threatening	Death

We were able to identify four sets of national or international adverse event severity grading criteria, with pediatric entries. The generic criteria for these grades subtly vary between all four sets.

- Division of Microbiology and Infectious Diseases Pediatric Toxicity Table (DMID) [6]: this draft consists of several, mainly laboratory, adverse events with even neonatal reference levels for different severity grades. No reference levels for preterms are given (currently a research topic within the CALIPER initiative [7]). Further very general adverse events are graded clinically (administration site problems, …). No specific neonatal diagnoses are included.

Grade 1	Grade 2	Grade 3	Grade 4	Grade 5
Mild	Moderate	Severe	Life threatening	Death

Salaets T, *et al. Arch Dis Child* 2019; 104:1167–1173. doi: 10.1136/archdischild-2019-317399

Transient or mild discomfort (<48h); no medical intervention/therapy required	Mild to moderate limitation in activity – some assistance may be needed; no or minimal medical intervention/therapy required	Marked limitation in activity, some assistance usually required; medical intervention/therapy needed, hospitalizations possible	Extreme limitation in activity, significant assistance required; significant medical intervention/therapy required, hospitalization or hospice care probable	Death

- Division of Acquired Immune Deficiency Syndrome (DAIDS) Table for Grading the Severity of Adult and Pediatric Adverse Events [8]**:** this list also includes some laboratory reference levels for different severity grades for neonates, however as mentioned above, this seems a little bit rash. Also some long term AE's are included (e.g. developmental delay). Again no specific neonatal diagnoses are included.

Grade 1	Grade 2	Grade 3	Grade 4
Mild	Moderate	Severe	Potentially life-threatening
Symptoms causing no or minimal interference with usual social & functional activities.	Symptoms causing greater than minimal interference with usual social & functional activities.	Symptoms causing inability to perform usual social & functional activities.	Symptoms causing inability to perform basic self-care functions. Medical or operative intervention indicated to prevent permanent impairment, persistent disability, or death.

- Common Terminology Criteria for Adverse Events (CTCAE) [9]: The CTCAE has been developed by the National Cancer Institute, and includes definitions and severity criteria for a wide range of clinical adverse events. Some severity grading criteria for long term outcomes (spasticity, growth suppression, gait disturbance, …) are included, but again criteria for most acute neonatal adverse events (except for neonatal death) are lacking. More information is given below.

Grade 1	Grade 2	Grade 3	Grade 4	Grade 5
Mild	Moderate	Severe	Life threatening	Death
Mild; asymptomatic or mild symptoms; clinical or diagnostic observations only; intervention not indicated	Moderate; minimal, local or non-invasive intervention indicated; limiting age-appropriate instrumental activities of daily living	Severe or medically significant but not immediately life-threatening; hospitalization or prolongation of hospitalization indicated; disabling; limiting self-care activities of daily living	Life-threatening consequences; urgent intervention indicated	Death related to AE

Salaets T, *et al. Arch Dis Child* 2019; 104:1167–1173. doi: 10.1136/archdischild-2019-317399

- FDA Toxicity Grading Scale for Healthy Adult and Adolescent Volunteers Enrolled in Preventative Vaccine Clinical Trials **[10]:** as the title suggests this document does not include neonatal adverse events. No generic definitions for the different grades are mentioned in this document.

Grade 1	Grade 2	Grade 3	Grade 4
Mild	Moderate	Severe	Potentially life-threatening

Currently, none of the existing systems contains specific neonatal adverse events. Furthermore, the generic severity definitions that are used, might not be easily applied to neonatal adverse events.

Q2: Are you aware of any other nationally or internationally accepted severity grading scales that might include neonatal (or at least pediatric) adverse events?

Answers: 55

Five respondents (9%) were aware of other systems. Five other respondents referred to other adverse event reporting systems in a comment on one of the other questions. After revision only one of these systems was an AE severity scale applicable to a research setting:

Suggestion	Editor's note
Australia, state based AE reporting system	Pharmacovigilance tool. Patients or doctors should report if the events caused various stages of discomfort (functional impairment), whether medical assistance was necessary (treatment) and if it recovered or not (outcome). No actual scale was given.
GAIA project	Global initiative (focus on LMIC) to improve safety assessment specifically after immunization in pregnancy, endorsed by the Brighton Collaboration. Appears to focus on strict case definitions (and diagnostic certainty), and less on a severity scale. However the suggested criteria appear very interesting and are globally supported. *(Bonhoeffer J et al, Vaccine 2016.)*
NICHD Pedtool	Peditools.org is a website that include risk, percentile, … calculator tools for the practicing pediatrician. It also includes a NICHD supported calculator for preterm birth outcomes. No severity scales available through this tool, however not sure if this is what the respondent suggested to use.
Du et al, ADR detection algorithm	Provides an algorithm to make the distinction between ADR and AE, but does not give guidance on severity classification. *(Du et al, J Clin Pharmacol 2013.)*
COG AE scoring tool	Not able to retrieve what this respondent meant.
WHO grades for severity	Not able to retrieve WHO-supported AE severity definitions.
DAIDS toxicity tool	Was already mentioned in the text.
Consequence scoring	Used for risk assessment in e.g. NHS trusts. Consists of a 5 point scale (from negligible to catastrophic) that can be evaluated at the patient's level, but also for organizational and population wide risks. Parameters in the scale are the need for medical intervention, time off work, prolongation of hospitalization, … Not designed for adverse event evaluation in a research setting. http://www.nrls.npsa.nhs.uk/EasySiteWeb/getresource.axd?AssetID=60149

Salaets T, *et al. Arch Dis Child* 2019; 104:1167–1173. doi: 10.1136/archdischild-2019-317399

CDISC	CDISC is a global, open, multidisciplinary, non-profit organization that has established standards to support the acquisition, exchange, submission and archive of clinical research data and metadata. The CDISC mission is to develop and support global, platform-independent data standards that enable information system interoperability to improve medical research and related areas of healthcare. CDISC focusses on coding of information for further analysis. Currently no severity grades for adverse events in neonates have been coded. In the future our grades could be added to CDISC.
ICD	International classification of diseases, no severity scale included.

Q3: Do you think we should use and apply the generic definitions of the different severity grades of one of the above mentioned systems for specific neonatal adverse events?

Answers: 54

The responses to this question were rather balanced. 57% (31 respondents) rejected the use of one of the existing systems. 43% (23 respondents) accepted to use one of the existing systems. Two respondents mentioned the CTCAE criteria, one respondent the DAIDS toxicity table as the most appropriate criteria to use in a neonatal setting.
Four respondents commented that we can start from one of the existing systems, but introduce minor modifications or add specifications on how to apply the criteria to neonates. Two respondents commented that the currents criteria in the existing systems do not seem to fit neonates.
One other respondent commented that it becomes confusing if multiple definitions (multiple factors?) are used. Three other respondents suggested to use another system: WHO grades, consequence scoring and NICHD ped tool. Their suggestions are reviewed above.

Q4: Do you think we should link severity grading criteria for neonatal adverse events with one of the existing systems? (Add neonatal adverse events to one of the existing systems in collaboration with its authors, for the purpose of generalization.) If yes, to which system?

Answers: 55

Again the response to this question is rather balanced, but surprisingly it is not in line with the previous question. Although a majority of respondents think we should not use the definitions of one of the existing systems, the majority (53% or 29 respondents) now suggests to add our entries to one of the existing systems instead of reinventing the wheel.
As 1 respondent pointed out this will depend on how close we stay to one of the existing systems. If our definitions or adaptations deviate too much from the original criteria, it will be difficult to liaise.
Five respondents commented that CTCAE would be the most ideal partner, while 3 commented that it would be best to liaise with the FDA toxicity table for healthy volunteers in vaccine trials. Another respondent suggested to also liaise the scale with CDISC. Three also referred to NICHD, 1 to WHO and 1 to ICD, however we are not aware of any AE severity scales supported by these organizations (reviewed above). Others were unsure.

Q5: Seriousness is not a synonym for severity. Do you think we should aim to parallel the generic definition of 'severe' as much as possible to the widely used definition of 'serious', so criteria can be used to help reporting both aspects?

Answers: 52

Salaets T, *et al. Arch Dis Child* 2019; 104:1167–1173. doi: 10.1136/archdischild-2019-317399

A large majority (77% or 40 respondents) of respondents think it should be best to parallel both definitions as much as possible, in order to help reporting both aspects.
Eight respondents commented that both aspects have a distinct meaning and thus the difference should remain clear. On the other hand, three respondents favor a harmonization of both terms. One respondent points at the various occasions where non-matching of the clinical assessment with the data, that companies and regulatory authorities process, occurs, to advocate a better coherence between both definitions.
One respondent also indicates that for some diagnoses the distinction between serious and severe will be wider than for others, another respondent illustrates this with IVH.
Three respondents also suggest that the definition of seriousness would benefit of some additions on how to apply these criteria to neonates/a unique system for neonates.

Defining severity in neonates: the development of generic criteria

On what base can we define severity in neonates?

Neonates are a specific population, with specific characteristics, making it difficult to define 'severity' with the same parameters as in adults and children. In this paragraph we make some suggestions of aspects of diseases that can be used as generic severity markers, and we discuss briefly their disadvantages.

Q6: Before reading the suggestions below: if we would construct a specific system for neonates which aspects of a disease would you use intuitively to grade its severity?

Answers: 50

As summarized by the editor:

**Categories**	**Respondents**
Long term consequences (actual effect on physical, mental and social development, need for post discharge care, ...)	16 (32%)
Immediate functional consequences (feeding difficulties, ...)	15 (30%)
Treatment	11 (22%)
Hospitalization and time of discharge	8 (16%)
Prognosis (potential effect on development)	6 (12%)
Supportive measures	5 (10%)
Respiratory status (oxygenation, FiO2, ...)	4 (8%)
Vital signs (HR, T, BP, ...)	4 (8%)
Mortality	4 (8%)
Clinical symptoms	2 (4%)
Imaging	2 (4%)
Economical cost	2 (4%)
Organ repercussions	2 (4%)
Genetics, brain damage, consciousness, laboratory results, repercussions on family, disease specific biomarkers and duration of the AE	All 1 (2%)

Salaets T, *et al. Arch Dis Child* 2019; 104:1167–1173. doi: 10.1136/archdischild-2019-317399

One respondent replied that multiple aspects should be used without further specification. One respondent replied that parameters are preferably quantifiable. Another also pointed out that the high baseline morbidity level should be taken in to account, and that the underlying condition should be considered.
One respondent replied to use one of the existing systems and one replied to use the FDA toxicity table (for healthy volunteers in vaccine trials). Some respondents also seem to have misunderstood the question and replied with mild-moderate-severe, ...

Immediate functional consequences

The main parameter in the above mentioned severity scales for adults and children is the effect of the adverse event on daily functioning at the moment of occurrence. Typical examples of activities of daily living (ADL) are self-feeding, personal hygiene and functional mobility. These activities can obviously not be used to define functional impairment in neonates. The effect of an adverse event on normal feeding behavior might be considered as a severity marker, but also in the 'normal' course of a preterm baby, without adverse events, feeding support is often necessary.

Q7: Do you think we should consider the immediate functional consequences of an adverse event as a parameter for severity? Impairment of which activities could be a good severity marker?

Answers: 52

A large majority of 81% (42 respondents) agreed to include immediate functional consequences. Two commented that it will be the only parameter available at the moment of grading.
In general two (related) types of immediate functional consequences are proposed by our respondents.

1. Changes in voluntary, conscious functions: oral feeding (5, 10%), voluntary movements and activity (3, 6%), crying pattern (1), social interactions (1), perception of pain (on a scale) (1), …
2. Disturbance of basal physiological processes: (spontaneous) breathing and oxygenation (12, 23%), blood pressure and perfusion (4, 8%), metabolic stability (4, 8%), consciousness and sleeping pattern (3, 6%), organ impairment (kidney, liver, …) (3, 6%), seizures (3, 6%), coagulopathy (2, 4%), enteral functioning (food tolerance, production of stools) (2, 4%), urine production (1), glycemia (1), … This aspect correlates very closely to the need for supportive measures discussed below.

One person underlined the need to specify the gestational age and postnatal ages at which certain parameters (e.g. feeding behavior) could be used. On the other hand three respondents suggested to use relative terms when talking about acute effects (a decline in abilities or physiological parameters).
Four respondents pointed out that it will be often difficult to link cause (the AE) and effect (decreased feeding behavior, …), and that the underlying condition may be a confounding factor.
Some people also commented that interventions (treatment or supportive measures) are the most reliable immediate consequences which can guide severity grading: this topic is discussed below. Other respondents commented on long term functional outcomes as being more important than the acute effect on the neonates functioning: also this topic is discussed below. One also commented that there is often no good relationship between acute effects and long term outcome, suggesting that long term outcome should have more weight than acute effects.

Treatment

The necessity of (invasive) treatment is another important marker of severity in the above mentioned scales. An important remark here is that often a wide variability of treatment protocols between centers exist (e.g. NEC, …). Defining severity based on treatment decisions could therefore result in a less standardized severity scale.

Salaets T, *et al. Arch Dis Child* 2019; 104:1167–1173. doi: 10.1136/archdischild-2019-317399

Q8: *Do you think we should consider the necessity and invasiveness of treatment for an adverse event as a parameter for severity? Why?*

Answers: 55

A large majority of 82% (45 respondents) agreed to include treatment decisions as a parameter in a neonatal severity scale.

Many respondents (15, 27%) comment that, despite the lack of standardization of treatment protocols, the necessity for treatment still reflects the severity of the adverse event and is thus an important factor for an AE severity scale. Ten respondents (18%) also commented that the treatment of the adverse event might impose additional risks for the neonate, and that thus can worsen (long term) outcomes. Four respondents pointed out that treatment is also related to economical cost of an adverse event and that this should be taken in to account. One respondent also commented that since treatment is an important factor in seriousness, we should also include it in a severity scale.

On the other hand four respondents warned for the lack of standardization between treatment protocols, and the bias that this might impose on safety data. (One of them suggested that a better approach would be to specify lab values (pH, …) at which treatment is initiated.) One respondent however points out that the lack of standardization is also not a problem for severity grading of adverse events in adults.

Five other respondents warned for the difficulty to link treatment (especially supportive measures) and adverse event: the initiation of certain treatments might also be due to the underlying condition of the neonate. Therefore indications of treatments should be well documented.

One respondent also pointed out that for some very severe adverse events no treatment might be possible.

Need for/prolongation of hospitalization

Also the need for hospitalization or prolongation of a current hospitalization is sometimes used as a severity marker (e.g. CTCAE generic criteria). However, preterm children might have intrinsically long hospitalizations even without significant comorbidity or adverse events. Even quite severe pathology along the route might not prolong the 'normal' hospitalization duration. Admission to an intensive care facility might be considered as marker for life threatening disease, however, in NICU-facilities not necessarily all patients are critically ill patients (some more in need of monitoring). Again, relying on these medical decisions could result in a less standardized severity scale.

Q9: *Do you think we should consider the need for/prolongation of hospitalization as a parameter for adverse event severity? Why?*

Answers: 55

A majority of 75% (41 respondents) agreed to include decisions regarding hospitalization as a parameter in a neonatal severity scale.

Five people commented that it would be good to include length of stay in a severity scale, since it is also included in widely accepted adult scales and in the definition of serious. Increased length of stay reflects well the severity of the causing AE, even though it is a (subjective) medical decision, said 5 respondents. Thirteen others pointed out that that the increased length of stay might on his turn have consequences that justify the use of hospitalization decisions as a severity marker. Six of those focused on the association of prolonged hospitalization with short (hospital infections, complications, …) and long term outcomes. Five of those also mentioned the high economical costs associated to prolonged hospitalisations. Five others pointed out that the length of stay has significant impact on patients and their families.

Salaets T, *et al. Arch Dis Child* 2019; 104:1167–1173. doi: 10.1136/archdischild-2019-317399

However many respondents (18), commented that there might be a lot of confounding factors that trouble the relation between AE severity and length of stay, questioning the feasibility of this marker. Frequently discussed confounding factors are the underlying condition of the child (preterm, comorbidities, …) and local differences between centers. A frequent comment was that preterm infants have intrinsically long hospitalisations and that even very severe AE's might not prolong them. Three respondents therefore explicitly suggested to use length of stay as a severity marker for term infants, but not for preterm neonates. Two others suggested to compare to local benchmarks adjusted for gestational age. One other suggested to focus on level of care changes (NICU versus neonatal care). One also pointed out that, at the occurrence of the adverse event, it is unforeseeable whether it will prolong hospitalization.

Supportive measures

Besides curative treatment, we could also consider the need for supportive measures (e.g. the need for invasive respiratory support) as a marker of severity, since it reflects respiratory failure. However, preterm babies have a relatively high baseline risk for respiratory support during their hospitalization. Therefore not all ventilated babies with adverse event X, are ventilated because of X. Again, the decision to install respiratory supportive measures remains to some extent subjective.

Q10: *Do you think we should consider the need for supportive measures as a parameter for adverse event severity? Why?*

Answers: 55

A large majority of 85% (47 respondents) agreed to include the need for supportive measures as a parameter in a neonatal severity scale.
Twelve respondents suggested to use the notion of increase or change in existing supportive measures (or level of care) as a parameter for severity. One suggested to focus on prolongation of the duration of supportive measures. One suggested to specify which measures should be taken in to account and which not, one suggested to only use the severe ones (start of ventilation, …). Two also claimed that this a severity marker that is objectively measurable. One however pointed out there will be large practice differences.
Three respondents thought supportive measures are important markers of severity because of their implications on quality of life, families and health care costs, while four mentioned the additional iatrogenic short and long term morbidities associated to these measures.
Eight expressed the concern that it can be difficult to distinguish whether supportive measures had to be increased due to the AE or the underlying condition, some of them stressed that only supportive measures related to the AE should be considered. Certainly in preterm infants this distinction will be challenging.
One respondent commented that it would be good to maintain consistency with severity scales for other populations, and thus supportive measures should not be included. Some people also pointed out throughout their comments that supportive measures should not be separated from the more general term 'treatment'. According to two other respondents primary treatment is more important, while supportive measures are indirect markers for severity.

Long term outcome

In a growing and developing population such as neonates, long term outcome is intuitively an important marker of adverse event severity. However in the immediate setting of a hospitalization, long term outcome of an adverse event (e.g. PVL) is not always so clear. Although associations between some degrees of IVH, PVL, ROP, … and long term neurodevelopmental outcomes have been found, it remains difficult to predict evolution in

Salaets T, *et al. Arch Dis Child* 2019; 104:1167–1173. doi: 10.1136/archdischild-2019-317399

individual cases. In reality, within the follow-up time of a study, we will have to deal with a prognosis instead of an actual outcome.

Q11: *Do you think we should consider long term outcome or prognosis as a parameter for adverse event severity? Why?*

Answers: 55

A majority of 73% (40 respondents) agreed to include long term outcome and or prognosis as a parameter in a neonatal severity scale. However the comments on this question are highly variable.
Basically in the comments of the respondents this issue is split up in two concepts: the actual long term outcome in the patient suffering from the AE, versus the population based prognosis of a certain AE.

Long term outcome

Thirteen respondents warned, as on previous questions, that it might be very difficult to link long term outcomes causally to the AE. Many confounding factors intervene, and preferably only truly related outcomes should be used to grade severity. Six respondents also mentioned that it is not easy to collect standardized long term follow-up data, and that the appropriate tools in well designed-follow-up studies should be used. More in general the need for good long term follow-up studies is emphasized by many more respondents throughout their comments. Since we are dealing with a developing population, long term outcomes are felt to be very important, by many respondents (regardless whether they think it should be included in a severity scale). Five respondents therefore added that the long term outcome is from a patient and family perspective probably the most relevant marker for severity.
Six people also commented that there will be temporal difficulties in using long term outcomes in a severity scales: AEs are currently reported in a transversal way (at occurrence or within 24h) and long term outcomes are not yet available then. Re-grading severity of adverse events after 2 years, to include follow-up data, seems unpractical, and thus these 6 people advocate not to include long-term outcomes in severity scale. On the other hand 1 respondent suggests to re-evaluate severity of all reported AEs at the end of follow-up. This issue is further discussed in one of the following questions.
As a solution to both issues (causal links and temporal difficulties), 1 respondent suggests to look at long term outcomes (cerebral palsy, visual impairment, …) as separate AE's.
Furthermore 1 thinks long term outcomes should be included to keep severity consistent with definitions of seriousness. One other feels that long term outcome is more a parameter of duration then of severity of an AE.

Prognosis

Eight respondents commented on the possibility to use prognosis as a marker for severity. Two of them resist to use prognosis as a severity marker (because of uncertainty at an individual level). One says using prognosis to grade severity will bias safety data: it will lead to safety data based on predictions instead of measured outcomes. On the other hand 2 respondents favor the use of prognosis as an important marker for severity and illustrate this with examples (acute kidney injury, BPD, …). The remaining 4 are more balanced. One is unsure on how to measure this, while three advise to only include prognosis if the likelihood of a bad outcome is high or if these surrogate measures are validated.

Q12: *Are there any other aspects of neonatal AE's, which could be good markers of severity?*

Answers: 40

Suggestions as summarized by the editor:

- Parental concerns and effect on parent-child interactions: 2x
- Secondary organ system injury or physiological derangement (AKI, ...): 3x

- Laboratory or imaging biomarkers: 2x
- Pain (scale)
- Duration of impairment

All other respondents replied previously discussed aspects or acknowledged that most were already covered.

Q13: *Which of the above mentioned severity markers should be the major component of a severity scale for neonatal adverse events?*

Answers: 43

As summarized by the editor:

**Severity marker**	**Respondents**
Immediate functional consequences	9 (21%)
Invasiveness and need for intervention	10 (23%)
Prolongation of hospitalisation	6 (14%)
Supportive measures	4 (9%)
Long term outcome or prognosis	19 (44%)

1 respondent replied to use biomarkers as a major component. 2 suggested that the major factor is pathology dependent.

Timing of the severity assessment in neonates

A very important (highly related) issue is **the time point at which severity will be assessed**. Especially for neurological problems (PVL, IVH, HIE, ROP), severity differs significantly between short (often asymptomatic) and long term (often very disabling). If one needs to grade an actual adverse event at the end of a study, it should be clear whether only immediate or also long term consequences will be taken in to account in the severity scale. There are different strategies to approach this issue: to assess overall AE severity "at a long term" at the end of the study, or to assess AE severity of typical neonatal problems at discharge (e.g. PVL) and to assess long term outcomes as separate AE's (e.g. cerebral palsy), or to …

Some important remarks:

- Study follow-up is not infinite, and thus it is possible that severe (developmental) consequences of a given adverse event only become apparent after the follow-up time.
- Although associations between some degrees of IVH, PVL, ROP, … and long term neurodevelopmental outcomes have been found, it remains difficult to predict evolution in individual cases. (Cfr. supra.)
- We can look to other severity grading sets to see how they tackle this problem. In the CTCAE criteria for example, several long term outcomes (e.g. gait disturbance, spasticity, …) are considered as separate AE's, and are not considered in the grading of their underlying adverse event (e.g. CVA, …). However this list mainly consists of adult, and some pediatric AE's. In those populations, in contrast to neonates, the concept of growing in to deficits is less important.

Q14: *When do you think severity of adverse events should be graded? At the occurrence of the adverse event? At discharge? At the end of the study follow-up time? Any other options?*

Answers: 54

Salaets T, *et al. Arch Dis Child* 2019; 104:1167–1173. doi: 10.1136/archdischild-2019-317399

Many people suggested to use multiple time points:

At occurrence	42 (78%)
At discharge	21 (39%)
At the end of study follow-up	20 (37%)

Some people suggested alternative options. Two people suggested as an alternative option that AEs should be graded at resolution, and thus that infants who suffer from an AE in a clinical trial should be flagged for follow-up until resolution of the AE. One suggested to decide on a specific evaluation period after the occurrence, but surely sooner than discharge. One other alternative was to specify developmental stages where long term follow-up needs to be performed.
Four respondents felt that the optimal time point of severity assessment is disease, drug and context-specific. Administration related AEs can be graded immediately, typical neonatal diagnoses (RDS, BPD, …) should be graded at discharge, while others like cerebral palsy will need long term follow-up.
Six respondents used this question to emphasize the importance of long term follow-up after clinical trial participation. One also proposes to use registries that can be used by different health care providers to collect data on the long term outcome of study participants. One also comments that it is important to explain the need for follow-up to parents.
Six others suggest the possibility of longitudinal assessments, where data collection starts at the occurrence of the AE, but where grading can be adapted in function of the course during and after hospitalization. On the other hand 3 people reported potential problems with this approach: the causal link between the actual long-term outcome and the AE might not be easy to make, there might be temporal problems with grading AEs at a distance of occurrence (no complete safety data available until end of follow-up, recall bias, …) and this probably will create inconsistent data unless all durations of follow-up are standardized.

Priority list for application of the generic criteria

Once this effort results in a generic severity scale for neonatal adverse events, we will proceed with the application of this scale on specific neonatal adverse events. This will result in a document that specifies what is understood to be a mild, moderate, severe or life-threatening version of a given adverse events. The use of this standard to collect safety data in the future will enhance the strength and comparability of these data.

We will not be able to find consensus on all possible neonatal adverse events. Therefore we would like to create a priority list, with adverse events that are most commonly reported in neonatal clinical trial safety data. Both acute events and long term outcomes can be included.

Please consider that laboratory adverse events, might be more difficult to address, since correct normal reference values are still lacking for many parameters (hemoglobin, creatinine, liver enzymes). There is still major ongoing work on this (e.g. CALIPER-initiative [7], Data Workgroup INC, …).

Q15: *Which adverse events should be included in the initial priority list for severity grading?*

Answers: 43

We received many suggestions on this question. All answers are listed here:

AE	Respondents
IVH	7 (16%)
NEC	7 (16%)

Salaets T, *et al. Arch Dis Child* 2019; 104:1167–1173. doi: 10.1136/archdischild-2019-317399

Hypotension	6 (14%)
BPD	5 (12%)
Cerebral palsy or motor impairment	4 (9%)
Seizures	4 (9%)
Sepsis	4 (9%)
ROP	3 (7%)
Cognitive impairment	3 (7%)
Acute kidney injury, PVL, apnea of prematurity, arrhythmia, persistent pulmonary hypertension, hypertension, extravasation, feeding intolerance	2 (5%)
Visual impairment, hearing impairment, psychiatric morbidity, spontaneous intestinal perforation, adrenal insufficiency, meningitis, hepatitis, need for intubation, encephalopathy, patent ductus arteriosus, pneumothorax, hyperglycemia, acidosis, toxic epidermal necrolysis, rash, reflux disease	1 (2%)

Others commented on this question by giving broader, less specific suggestions:
Six respondents suggested to include health care associated conditions (medication errors, delay of care, catheter related problems, …). Four responded that all events with chronic impact on organ or system functioning and growth will need priority. One also suggested to include prenatal and delivery related AEs.
Three wanted to include mainly cardiorespiratory AE's, while 3 others preferred to start with neurological AEs. Two simply said to grade the most common neonatal diagnoses. One suggested to check the adverse event list of a range of clinical trials in preterm neonates.
One other suggested to base our priority list on recent and future INC output (Clin Pharm white paper, Seizures protocol, …).
Some people seem also to have misunderstood the question as they proposed to preferentially grade unexpected AEs, ADRs or specific AEs depending on the clinical trial.

Q16: *Do you have any other suggestions or comments on this subject?*

Most respondents did not have any comments.
One was wondering which would be the age-limits for use of our final adverse event severity criteria, and whether there would be a distinction based on post-menstrual age or post-natal age.
Three people commented that there are other issues in AE reporting, which need more attention than severity. One thought we should focus on seriousness, one on guidelines on ADR versus AE, and one on the distinction between AEs and secondary outcome measures.
One last respondent suggested that it would be good to integrate our criteria in to the CDISC data standards (cfr. above).

References

Salaets T, *et al. Arch Dis Child* 2019; 104:1167–1173. doi: 10.1136/archdischild-2019-317399

1. *EVERREST - Regenerative Medicine Clinical Trials*. 2016; Available from: http://everrest-fp7.eu/.
2. McMillan, S.S., M. King, and M.P. Tully, *How to use the nominal group and Delphi techniques.* Int J Clin Pharm, 2016. **38**(3): p. 655-62.
3. Kahn, M.G., et al., *Building a common pediatric research terminology for accelerating child health research.* Pediatrics, 2014. **133**(3): p. 516-25.
4. *Pediatric Terminology Files*. 2016; Available from: https://evs.nci.nih.gov/ftp1/NICHD/About.html.
5. *Clinical Safety Data Management: Definitions and Standards for Expedited Reporting*. 2016; Available from: http://www.ich.org/fileadmin/Public_Web_Site/ICH_Products/Guidelines/Efficacy/E2A/Step4/E2A_Guideline.pdf.
6. *Division of Microbiology and Infectious Diseases (DMID) Pediatric Toxicity Tables*. 2016; Available from: https://www.niaid.nih.gov/sites/default/files/documents/dmidpedtox.pdf.
7. *CALIPER Project*. [text] 2016; Available from: http://www.sickkids.ca/caliperproject/index.html.
8. *Division of AIDS Table for Grading the Severity of Adult and Pediatric Adverse Events*. 2016; Available from: http://ucdmc.ucdavis.edu/clinicaltrials/StudyTools/Documents/DAIDS_AE_GradingTable_FinalDec2004.pdf.
9. *Common Terminology Criteria for Adverse Events*. 2016; Available from: https://evs.nci.nih.gov/ftp1/CTCAE/CTCAE_4.03_2010-06-14_QuickReference_5x7.pdf.
10. *Toxicity Grading Scale for Healthy Adult and Adolescent Volunteers Enrolled in Preventive Vaccine Clinical Trials*. 2016; Available from: http://www.fda.gov/downloads/BiologicsBloodVaccines/GuidanceComplianceRegulatoryInformation/Guidances/Vaccines/ucm091977.pdf.

Salaets T, *et al. Arch Dis Child* 2019; 104:1167–1173. doi: 10.1136/archdischild-2019-317399
