# Supplementary material for: Development of a neonatal adverse event severity scale through a Delphi consensus approach
Source: Arch Dis Child. 2019 Sep 19;104(12):1167–73. doi: 10.1136/archdischild-2019-317399 (PMC6943241; doi:10.1136/archdischild-2019-317399)
Supplement: Supplementary data [file archdischild-2019-317399supp004.pdf]

Appendix 4

Report of survey 2

Introduction and methods

Based on the results of a first stakeholder survey a proposal for generic severity criteria was sent to the stakeholders in this second survey. The different parts of this proposal were supported by a summary of the relevant results of the first survey *(in italics)*, including opposing views. It was presented to the stakeholders as given below.

This proposal was reviewed by 36 stakeholders, from all relevant stakeholder groups.

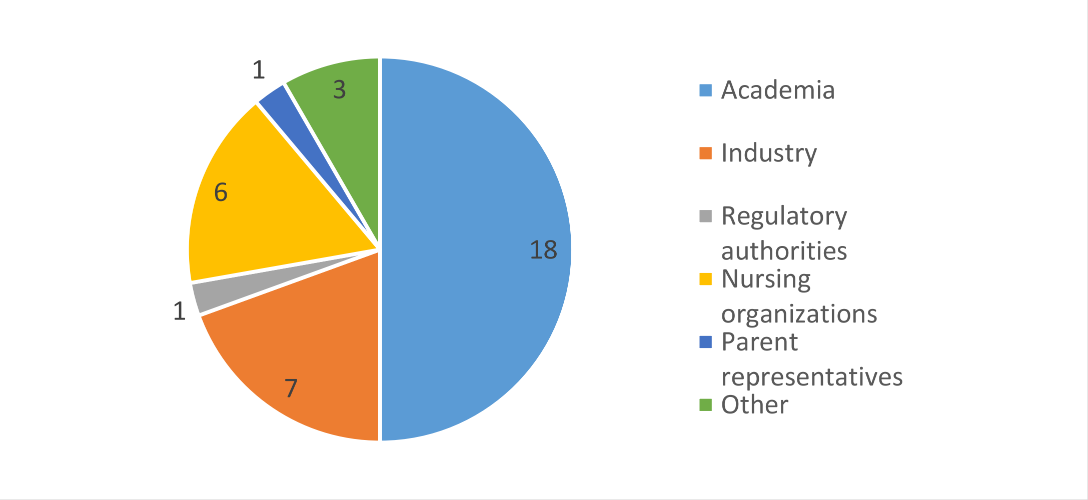

Figure 1. Stakeholders that provided input in 2nd survey

In two questions respondents were asked whether or not they agreed with the framework and content of this proposal for generic severity criteria. The results are summarized in the blue boxes.

Framework

Case definitions

Before giving criteria for severity of a certain AE in our final severity table, we need to describe the case definition of that certain AE. In an ideal scenario, these definitions are linked to internationally accepted definition registers. We will **use the existing NICHD Pediatric Adverse Events Terminology**, which was mapped to MedDRA previously and is accessible through NCI vocabulary services, as a pragmatic starting point for AE definitions. [1, 2]

96% (53) of respondents agreed to use the NICHD Pediatric Adverse Events Terminology. Only two respondents objected. Two respondents commented that many of the definitions are pathology or etiology based, and this is often hard to use in a clinical setting. One respondent commented that some definitions (BPD) are outdated.

Linking and adherence to existing systems

Other severity grading systems exist in other populations. For the purpose of generalization, we suggest to add neonatal AE severity grades to an existing toxicity table in collaboration with its authors. Because of the its wide spread use, its coverage of a very wide range of adverse events in other populations and the slight preference of our neonatal stakeholder panel, we propose to **liaise with the CTCAE [3]**. Our goal could be to add a neonatal chapter to their toxicity table.

Salaets T, *et al. Arch Dis Child* 2019; 104:1167–1173. doi: 10.1136/archdischild-2019-317399

Proposal for generic criteria of neonatal AE severity

A small majority of respondents (53%, 29) recommended to add neonatal AE entries to one of the existing systems. Five respondents commented that CTCAE would be the most ideal partner, while 3 commented that it would be best to liaise with the FDA toxicity table for healthy volunteers in vaccine trials. In total 10 respondents suggested other systems (cfr. full report), however none of them is a formal AE severity scale.
One respondents warned that if our definitions or adaptations deviate too much from the original criteria, it might be difficult to liaise.

The definitions and wording of the generic CTCAE severity grades, does not seem applicable to the specificities of neonatal populations (as do also the definitions of the other existing systems). The generic criteria of CTCAE for the general population are shown in table 1.

Grade 1	Grade 2	Grade 3	Grade 4	Grade 5
Mild	Moderate	Severe	Life threatening	Death
Mild; asymptomatic or mild symptoms; clinical or diagnostic observations only; intervention not indicated	Moderate; minimal, local or non-invasive intervention indicated; limiting age-appropriate instrumental activities of daily living	Severe or medically significant but not immediately life-threatening; hospitalization or prolongation of hospitalization indicated; disabling; limiting self-care activities of daily living	Life-threatening consequences; urgent intervention indicated	Death related to AE

Table 1. Generic criteria of AE severity, according to CTCAE

We propose to **add some specifications and make minor adaptations** to these existing generic criteria, in order to make them applicable to 'a generic neonatal AE', in line with the following principles.

Grade 1	Grade 2	Grade 3	Grade 4	Grade 5
Mild	Moderate	Severe	Life threatening	Death
A change in condition that we can record, but does not adversely affect the baby	A change in condition which has an impact but which the baby can tolerate without a change in care	A change in the condition of the baby requiring a significant care changes	A change in condition that is life threatening	Death related to AE

Table 2. Principles for a neonatal AE severity scale

A majority of 57% (31) of the respondents rejected the use and application of one of the existing systems for neonatal AEs (in their current form and wording). Some commented that these definitions do not seem applicable to neonates. Four respondents commented that we could start from one of the existing systems, but introduce minor modifications or add specifications on how to apply the criteria to neonates.

Seriousness versus severity

In safety event reporting, a 'serious AE' is not necessarily a 'severe AE' and vice versa. Reporting seriousness is a regulatory obligation and the definition of 'serious' is internationally accepted in the ICH Tripartite Guidelines [4].

A serious AE is any untoward medical occurrence that:

- results in death
- is life-threatening (The term "life-threatening" in the definition of "serious" refers to an event in which the patient was at risk of death at the time of the event; it does not refer to an event which hypothetically might have caused death if it were more severe).

Salaets T, *et al. Arch Dis Child* 2019; 104:1167–1173. doi: 10.1136/archdischild-2019-317399

- requires inpatient hospitalization or prolongation of existing hospitalization,
- results in persistent or significant disability/incapacity, or
- is a congenital anomaly/birth defect.

Table 3. Definition of a serious AE, ICH Tripartite Guidelines

In contrast, severity grading is used to enrich safety data by adding a label referring to the clinical relevance of the AE. It is thus a different concept, however in most adult severity scales all AEs graded as severe (or higher) seem to match the definition of 'serious' to some extent. **We suggest, despite the conceptual difference, to maintain this parallel between the practical meaning of 'severity' in neonates with the meaning of 'seriousness'.** Pragmatically, our neonatal severity scale can thus help researchers to make the distinction between AE and SAE (and thus standardize also this distinction), however this cannot be a strict recommendation.

A large majority (77%, 40) of respondents think it should be best to parallel both definitions as much as possible, in order to help reporting both aspects. One respondent points at the various occasions where non-matching of the clinical assessment with the data that companies and regulatory authorities process, occurs, to advocate a better coherence between both definitions. On the other hand eight respondents commented that both aspects have a distinct meaning and thus the difference should remain clear.

Timing of severity grading

We think the severity of AEs should be graded within a short time after occurrence. We think grading at the end of follow-up (or longitudinal re-assessments) will create practical problems: unclear links to the AE, recall bias, … This has implications on the possibility to use long term outcome as a formal marker of severity, however does not mean that we do not value long term follow-up (cfr. infra).

A majority of our stakeholder panel suggest to grade severity at occurrence (42, 78%). 39% (21) suggests to grade at discharge, and 37% (20) to grade at the end of follow-up. Some people suggested multiple time points. Some people suggested alternative options: at resolution (infants who suffer from an AE in a clinical trial should be flagged for follow-up until resolution of the AE), a specific evaluation period after the occurrence, but surely sooner than discharge or at specific developmental stages.
Four respondents felt that the optimal time point of severity assessment is disease, drug and context-specific. Six others suggest the possibility of longitudinal assessments, where data collection starts at the occurrence of the AE, but where grading can be adapted in function of the course during and after hospitalization. One also proposes to use registries that can be used by different health care providers to collect data on the long term outcome of study participants. On the other hand 3 people reported potential problems with the longitudinal approach: the causal link between the actual long-term outcome and the AE might not be easy to make, there might be temporal problems with grading AEs at a distance of occurrence (no complete safety data available until end of follow-up, recall bias, …) and this probably will create inconsistent data unless all durations of follow-up are standardized.

Age limits

We think we should provide some guidance on when to apply the specific neonatal adverse event severity criteria. **We propose to limit the use of these neonatal criteria to babies up to 44 weeks of gestational age**.

One respondent was wondering which would be the age-limits for use of our final adverse event severity criteria, and whether there would be a distinction based on post-menstrual age or post-natal age.

Q1: Can you agree with the framework of this proposal considering the arguments that have been provided by the responses from the stakeholder panel? If not, with which aspect (case definitions, linking to CTCAE, modifying the adult criteria, paralleling seriousness, timing of severity grading or age limits) do you disagree? Why? And how would you propose to change this aspect?

69% of respondents agreed with this framework. All others could agree but had comments.

Proposal for generic criteria of neonatal AE severity

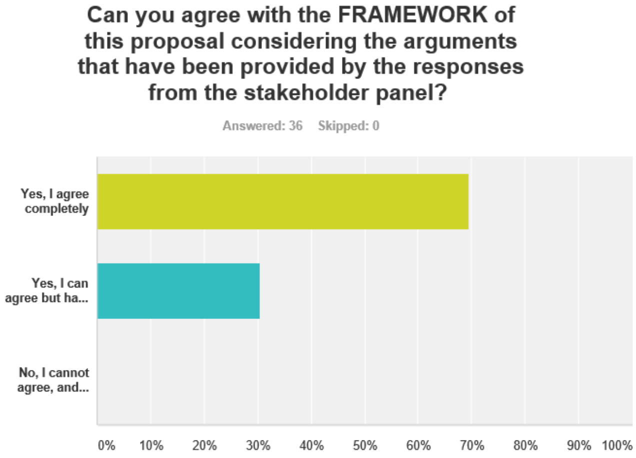

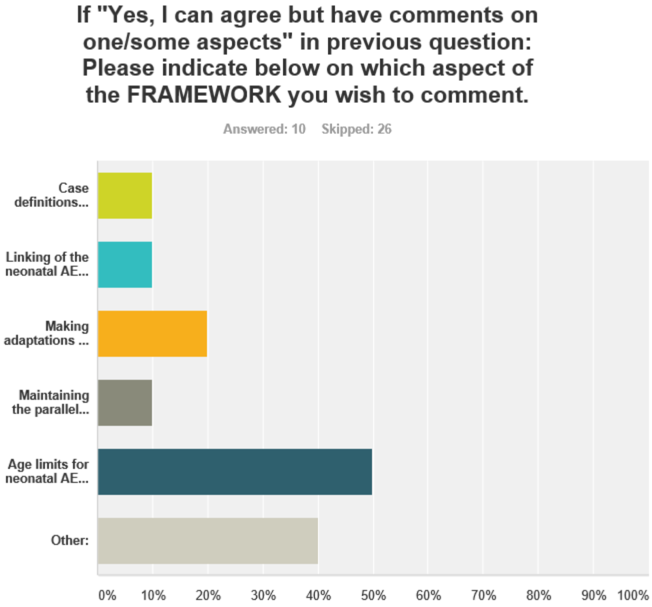

Comments received:

- Case definitions:
  - Many case definitions are undergoing revisions: how will we stay up to date with these changes?
- Adjusting the CTCAE criteria to fit for neonates:
  - One respondent prefers to start de novo for neonates, but can agree with this.
- Seriousness vs severity:
  - Despite standardization medical judgment will be required to define both serious and severe.
  - Clarify to the group the difference in purpose of serious and severe.
  - Change the definition of serious in the document: add 'medical important event'.
- Age limit at 44weeks postmenstrual age:
  - Do we want to limit the use of this criteria to babies enrolled before 44wk PMA? Or to, maybe earlier enrolled babies, once they become 44wk? (3)
  - There is no alternative for 45wk babies (or other infants). (2)
  - Can we go up to 3 – 6 months: Neonatal and Young Infant Adverse Event Severity Scale?
- Other: timing of severity assessment:
  - We should specify what short time after occurrence means? (3)

Salaets T, *et al. Arch Dis Child* 2019; 104:1167–1173. doi: 10.1136/archdischild-2019-317399

Proposal for generic criteria of neonatal AE severity

- Depends on the AE: some are appropriate to grade at occurrence, while others should be graded at discharge or even later. (2)

Generic criteria

Immediate consequences

We think that the **immediate functional consequences of an AE are important markers of severity, and thus should be included in our severity scale**. However current adult definitions (instrumental activities of daily life, activities of daily life, …) are difficult to apply in neonates, and **need modification or specification**. The stakeholder panel suggested to look at both voluntary/conscious functions and basal physiological processes. We emphasize that only consequences related to the AE should be considered.

	Grade 1	Grade 2	Grade 3	Grade 4	Grade 5
	Mild	Moderate	Severe	Life threatening	Death
CTCAE scale	asymptomatic or mild symptoms; clinical or diagnostic observations only	limiting age-appropriate instrumental activities of daily living*	disabling; limiting self-care activities of daily living**	Life-threatening consequences	
*Instrumental ADL refer to preparing meals, shopping for groceries or clothes, using the telephone, managing money, etc.<br>**Self care ADL refer to bathing, dressing and undressing, feeding self, using the toilet, taking medications, and not bedridden.					
Proposal neonatal scale	asymptomatic or mild symptoms; clinical or diagnostic observations only; no impairment of normal age-appropriate behavior*	resulting in limited impairment of normal age-appropriate behavior*	resulting in significant impairment of normal age-appropriate behavior* and/or limited disturbances of basal physiological processes**	life-threatening consequences	
*Age-appropriate behavior refers to oral feeding behavior, voluntary movements and activity, crying pattern, social interactions and perception of pain appropriate for the gestational and postnatal age of the neonate.<br>**Basal physiological processes refer to oxygenation, ventilation, tissue perfusion, metabolic stability.					

Table 4. Proposal for immediate functional consequences as a marker for neonatal severity

A large majority of 81% (42 respondents) agreed to include immediate functional consequences. However four respondents pointed out that it will be often difficult to link cause (the AE) and effect (decreased feeding behavior, …), and that the underlying condition may be a confounding factor.
In general two (related) types of immediate functional consequences are proposed by the stakeholder panel:

1. *Changes in voluntary, conscious functions: oral feeding (5, 10%), voluntary movements and activity (3, 6%), crying pattern (1), social interactions (1), perception of pain (on a scale) (1), …*
2. *Disturbance of basal physiological processes: (spontaneous) breathing and oxygenation (12, 23%), blood pressure and perfusion (4, 8%), metabolic stability (4, 8%), consciousness and sleeping pattern (3, 6%), organ impairment (kidney, liver, …) (3, 6%), seizures (3, 6%), coagulopathy (2, 4%), enteral functioning (food tolerance, production of stools) (2, 4%), urine production (1), glycemia (1), … This aspect correlates very closely to the need for supportive measures discussed below.*

Salaets T, *et al. Arch Dis Child* 2019; 104:1167–1173. doi: 10.1136/archdischild-2019-317399

Proposal for generic criteria of neonatal AE severity

One person underlined the need to specify the gestational and postnatal ages at which certain parameters (e.g. feeding behavior) can be used.

Interventions

Despite the lack of standardized treatment protocols, we think that the need for treatment very well reflects the severity of the AE. **We propose to include the necessity of treatment in the neonatal severity scale, in the same way as it is included in severity scales for other populations, and to include also supportive measures here.** We suggest to consider both primary and supportive treatment, but only when these are clearly initiated to treat the AE.

	**Grade 1**	**Grade 2**	**Grade 3**	**Grade 4**	**Grade 5**
	Mild	Moderate	Severe	Life threatening	Death
CTCAE scale		minimal, local or non-invasive intervention indicated	*(not specified in CTCAE)*	urgent intervention indicated	
Proposal neonatal scale	no intervention indicated	minimal, local or non-invasive intervention indicated	significant medical/surgical intervention indicated, including non-invasive supportive measures	urgent medical/surgical intervention indicated, including invasive supportive measures	

Table 5. Proposal for treatment as a marker for neonatal severity

A large majority of 82% (45 respondents) agreed to include treatment decisions as a parameter in a neonatal severity scale.
Many respondents (15, 27%) comment that, despite the lack of standardization of treatment protocols, the necessity for treatment still reflects the severity of the adverse event and is thus an important factor for an AE severity scale. Ten respondents (18%) also commented that the treatment of the adverse event might impose additional risks for the neonate, and that thus can worsen (long term) outcomes. Four respondents pointed out that treatment is also related to economical cost of an adverse event and that this should be taken in to account. One respondent also commented that since treatment is an important factor in seriousness, we should also include it in a severity scale.
On the other hand four respondents warned for the lack of standardization between treatment protocols, and the bias that this might impose on safety data. One respondent however pointed out that the lack of standardization is also not a problem for severity grading of adverse events in adults.

A large majority of 85% (47 respondents) agreed to include the need for supportive measures as a parameter in a neonatal severity scale.
Three respondents thought supportive measures are important markers of severity because of their implications on quality of life, families and health care costs, while four mentioned the associated iatrogenic short and long term risks. Two also claimed that this is a severity marker that is objectively measurable. One however pointed out there will be large practice differences.
Eight expressed the concern that it can be difficult to distinguish whether supportive measures had to be increased due to the AE or the underlying condition, some of them stressed that only supportive measures related to the AE should be considered. Certainly in preterm infants this distinction will be challenging. Twelve respondents suggested to use the notion of increase or change in existing supportive measures (or level of care) as a parameter for severity.
One respondent commented that it would be good to maintain consistency with severity scales for other populations, and thus supportive measures should not be included. Some people also pointed out throughout their comments that supportive measures should not be separated from the more general term 'treatment'.

Salaets T, *et al. Arch Dis Child* 2019; 104:1167–1173. doi: 10.1136/archdischild-2019-317399

Proposal for generic criteria of neonatal AE severity

Hospitalization

Following the suggestions from the stakeholder panel, we would like to **maintain hospitalization as a severity marker**. We feel that this will be still very useful for term neonates, and also in selected preterm cases (for instance near the end of hospitalization). We think duration and prolongation of hospitalization is probably not a good marker of severity of AEs occurring early in the admission of preterm neonates, however then we can still use interventions and functional consequences to grade these AEs. Considering level of care changes in this setting has been suggested, but will likely overlap with the need for treatment and supportive measures. Again, only hospitalizations that are clearly linked to the AE should be taken in to account for severity grading.

	**Grade 1**	**Grade 2**	**Grade 3**	**Grade 4**	**Grade 5**
	Mild	Moderate	Severe	Life threatening	Death
CTCAE scale			hospitalization or prolongation of hospitalization indicated		
Proposal for neonates			hospitalization or prolongation of hospitalization indicated		

Table 6. Proposal for treatment as a marker for neonatal severity

A majority of 75% (41 respondents) agreed to include decisions regarding hospitalization as a parameter in a neonatal severity scale.
Five people commented that it would be good to include length of stay in a severity scale, since it is also included in widely accepted adult scales and in the definition of serious. Thirteen others pointed out that that the increased length of stay might on his turn have consequences that justify the use of hospitalization decisions as a severity marker (related to short (hospital infections, complications, …) and long term outcomes, high economical costs, significant impact on patients and their families).
However many respondents (18), commented that there might be a lot of confounding factors (prematurity, underlying conditions, local customs, …) that trouble the relation between AE severity and length of stay. A frequent comment was that preterm infants have intrinsically long hospitalizations and that even very severe AE's might not prolong them. Three respondents therefore explicitly suggested to use length of stay as a severity marker for term infants, but not for preterm neonates. Two others suggested to compare to local benchmarks adjusted for gestational age. One other suggested to focus on level of care changes (NICU versus neonatal care).

Long term outcome and prognosis

Long term outcome is felt to be the most important factor that determines the severity of an adverse event in a neonatal population. All interventions and AEs can potentially harm long term outcome, more than in any other population. The importance of long term outcomes and follow-up is reflected in the responses we received from our stakeholder panel, and has been discussed in previous INC-efforts [5]. However, out of the large variety of comments we received on this topic, we understand **long term outcome is not feasible to use in a transversal severity scale**, because of temporal and practical difficulties and because of a the uncertain causal link between AE and outcome. Some people suggested to use population based prognosis as a proxy for long term outcome, however this might bias safety data towards expectations and predictions that are not necessarily true in individual cases. We propose therefore to **emphasize the importance of long term outcomes by including specific long term outcome events as separate AEs in the severity table** (motor, visual, cognitive, … impairment).

	**Grade 1**	**Grade 2**	**Grade 3**	**Grade 4**	**Grade 5**
	Mild	Moderate	Severe	Life threatening	Death
CTCAE scale					

Proposal for generic criteria of neonatal AE severity

Proposal for neonates					

Table 7. Proposal for prognosis/long term outcome as a marker for neonatal severity

A majority of 73% (40 respondents) agreed to include long term outcome and or prognosis as a parameter in a neonatal severity scale. Importantly, when we asked the stakeholder panel (before going through the questionnaire) which parameters they intuitively would include in a neonatal severity scale, long term consequences are the most popular suggestion (32%, 16). Prognosis was the 5th suggestion (12%, 6). Also, when we asked the stakeholder panel at the end of the questionnaire which of the discussed parameters should be the most important one in a severity scale, long term outcome and prognosis are valued the most (44%, 19), followed by treatment (23%, 10) and immediate consequences (21%, 9).
However this parameters gets less support when compared to the previous parameters, and the comments on this question indicate a strong interindividual disagreement.
Long term outcome
Thirteen respondents warned, as on previous questions, that it might be very difficult to link long term outcomes causally to the AE. Many confounding factors intervene, and preferably only truly related outcomes should be used to grade severity. Six respondents also mentioned that it is not easy to collect standardized long term follow-up data, and that the appropriate tools in well designed-follow-up studies should be used. More in general the need for good long term follow-up studies is emphasized by many more respondents throughout their comments. Since we are dealing with a developing population, long term outcomes are felt to be very important, by many respondents (regardless whether they think it should be included in a severity scale). Five respondents therefore added that the long term outcome is from a patient and family perspective probably the most relevant marker for severity.
Six people also commented that there will be temporal difficulties in using long term outcomes in a severity scales: AEs are currently reported in a transversal way (at occurrence or within 24h) and long term outcomes are not yet available then. Re-grading severity of adverse events after 2 years, to include follow-up data, seems unpractical, and thus these 6 people advocate not to include long-term outcomes in severity scale. On the other hand 1 respondent suggests to re-evaluate severity of all reported AEs at the end of follow-up.
As a solution to both issues (causal links and temporal difficulties), 1 respondent suggests to look at long term outcomes (cerebral palsy, visual impairment, …) as separate AE's.
Furthermore 1 thinks long term outcomes should be included to keep severity consistent with definitions of seriousness.
Prognosis
Eight respondents commented on the possibility to use prognosis as a marker for severity. Two of them resist to use prognosis as a severity marker (because of uncertainty at an individual level). One says using prognosis to grade severity will bias safety data: it will lead to safety data based on predictions instead of measured outcomes. On the other hand 2 respondents favor the use of prognosis as an important marker for severity and illustrate this with examples (acute kidney injury, BPD, …). The remaining 4 are more balanced. One is unsure on how to measure this, while three advise to only include prognosis if the likelihood of a bad outcome is high or if these surrogate measures are validated.

Other parameters for a severity scale, as suggested by the stakeholder panel, were parental concerns and effect on parent-child interactions, secondary organ system injury or physiological derangement (AKI, ...), laboratory or imaging biomarkers, pain (scale) and duration of impairment.

Synthesis

Our proposition is thus summarized in table 6, and is compliant with the wish to grade the severity of an adverse event timely after occurrence and the wish to maintain the parallel between the definitions of severity and seriousness.

Grade 1	Grade 2	Grade 3	Grade 4	Grade 5
Mild	Moderate	Severe	Life threatening	Death

Proposal for generic criteria of neonatal AE severity

Mild; asymptomatic or mild symptoms; clinical or diagnostic observations only; no impairment of normal age-appropriate behavior*	Moderate; minimal, local or non-invasive intervention indicated; resulting in limited impairment of normal age-appropriate behavior*	Severe or medically significant but not immediately life-threatening; significant medical/surgical intervention indicated, including non-invasive supportive measures; hospitalization or prolongation of hospitalization indicated; resulting in significant impairment of normal age-appropriate behavior* and/or limited disturbances of basal physiological processes**	life-threatening consequences; urgent medical/surgical intervention indicated, including invasive supportive measures	Death related to AE
*Age-appropriate behavior refers to oral feeding behavior, voluntary movements and activity, crying pattern, social interactions and perception of pain.<br>**Basal physiological processes refer to oxygenation, ventilation, tissue perfusion, metabolic stability and organ functioning.				

Table 8. Proposal for severity criteria for a generic neonatal AE

Q2: Can you agree with the criteria in this proposal considering the arguments that have been provided by the responses from the stakeholder panel? If not, with which parameter (immediate functional consequences, interventions, hospitalizations or long term outcome) do you disagree? Why? And how would you propose to change this aspect?

72% of respondents agreed with this proposal without comments. Only 1 respondent could not agree with this proposal. All others could agree but had comments.

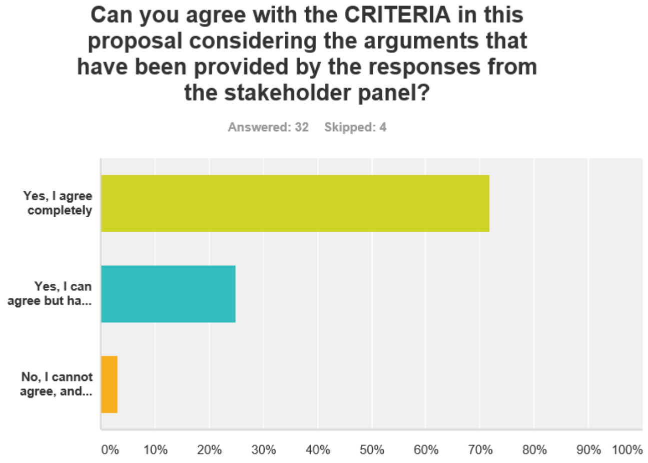

Salaets T, *et al. Arch Dis Child* 2019; 104:1167–1173. doi: 10.1136/archdischild-2019-317399

Proposal for generic criteria of neonatal AE severity

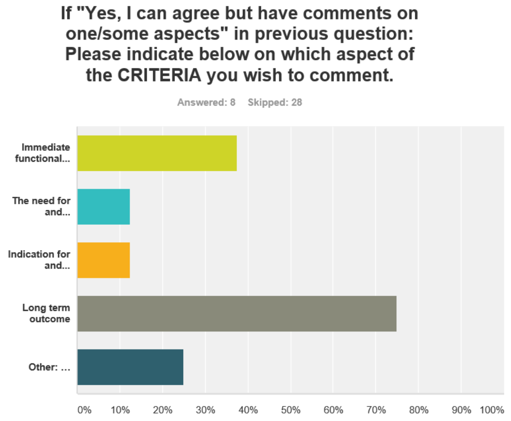

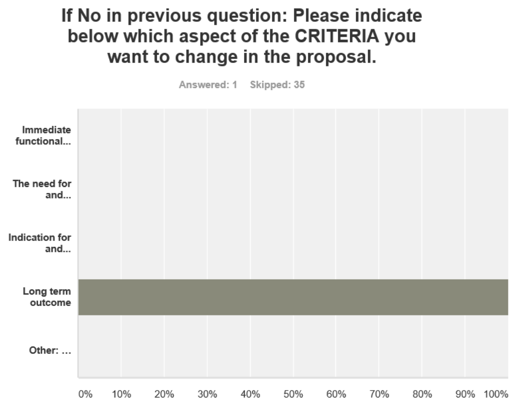

Comments:

- Immediate functional consequences:
    - Disturbance of basal physiological processes is open to wide interpretation (even with specifications)
    - These criteria might provide data that are not useful or interpretable
    - Might be difficult to link AE and consequence (confounding factors): maybe word 'possibly resulting in ...' and include a statement that grading functional consequences is of lesser significance than grading based on interventions.
- Need for hospitalization:
    - The difference in gestational age will affect length of hospitalization. There are no good bench marks for very low birth weight infants.
- Interventions:
    - We might need to define what significant treatment is: is a short period of antibiotics significant? How much change in ventilatory support?
    - Make sure to only look at changes in supportive measures related to the AE (confounding factors, ...).
- Long term outcome and prognosis:
    - Often it is very difficult to link long term outcome with AE (many confounding factors) (3)
    - It seems impractical to wait for long term outcome before grading severity (2), e.g. infants that are lost to follow-up, the need of re-assessments, ...
    - Maybe timepoint of resolution af an AE could be interesting (with guidance on how to define endpoints in AEs)
    - Look at long term outcome regardless of its etiology.
    - If long term outcome is included, length of follow-up and timing of grading should be standardized.
- Other:
    - Important to emphasize that many symptoms might go unnoticed in neonates (e.g. nausea will only be noticed if actual vomiting occurs)
    - Consider an education module to this piece to help investigators reporting AEs
    - The scale is very general which might be strength but also a weakness

Conclusion

Most respondents agreed with the proposed generic severity criteria and the framework for a neonatal AE severity scale. These results and all comments will be discussed in a face-to-face meeting on 27th of March to define final neonatal generic severity criteria.

References

Proposal for generic criteria of neonatal AE severity

1. *Pediatric Terminology Files*. 2016; Available from: https://evs.nci.nih.gov/ftp1/NICHD/About.html.
2. Gipson, D.S., et al., *Development of a Pediatric Adverse Events Terminology.* Pediatrics, 2017. **139**(1).
3. *Common Terminology Criteria for Adverse Events*. 2016; Available from: https://evs.nci.nih.gov/ftp1/CTCAE/CTCAE_4.03_2010-06-14_QuickReference_5x7.pdf.
4. *ICH harmonized tripartite guideline: Guideline for Good Clinical Practice.* J Postgrad Med, 2001. **47**(1): p. 45-50.
5. Ward, R.M., et al., *Safety, Dosing, and Pharmaceutical Quality for Studies that Evaluate Medicinal Products (including Biological Products) in Neonates.* Pediatric Research, 2016.
