# Supplementary material for: Development of a neonatal adverse event severity scale through a Delphi consensus approach
Source: Arch Dis Child. 2019 Sep 19;104(12):1167–73. doi: 10.1136/archdischild-2019-317399 (PMC6943241; doi:10.1136/archdischild-2019-317399)
Supplement: Supplementary data [file archdischild-2019-317399supp005.pdf]

Appendix 5

Report of survey 3

Introduction

This **document** is a final draft for a Neonatal Adverse Event Severity Scale. The generic scale has been developed based on the feedback of +50 stakeholders in a Delphi survey process. Based on these generic criteria, severity criteria for specific, and relatively common, neonatal adverse events were drafted. Criteria were discussed and proposed in Bethesda on April 11th during the Clin Pharm WG Meeting, by 43 experts from industry, regulatory authorities and academia. Consequently 51 experts from the same categories reviewed these criteria through a web survey (survey 3), resulting in the following document. In this document we summarized all comments. Disagreement is visualized on the following scale:

0-10%	10.1-20%	20.1-30%	30.1-40%	40.1-50%

We chose not to adapt the gradings of criteria with 20% or less disagreement, for all others adaptations have been worded. For each AE the original scale has been visualized, then the comments, and then the adapted scale (changes in red).

We did not provide a reason why it was or was not considered for every individual comment, but want to answer some general comments that we came across during the whole survey:

- An adverse event is defined as **any untoward medical occurrence** in a patient or clinical investigation subject administered a pharmaceutical product and which does not necessarily have a causal relationship with this treatment (ICH-GCP). The question whether it can be considered as related to the study drug or not is immaterial. With this tool we want to provide a framework by which research nurses/assistant can grade the severity of any event that occurred in a study patient.
- We aim to provide a framework to categorize the data that is available, but **do not give guidance on what should be reported** and what not. We agree that some mild AE definitions are probably too mild to get relevant information. Guidance on what to report should typically be given in the study protocol. This tool however helps by providing standard severity grades that can be used as cut-offs for reporting.
- It speaks to itself that **only changes from baseline conditions** should be considered. In a baby having a NG tube from the start, having a NG tube can't be considered as a severity criteria for a certain AE.
- It is not the task of our work group to provide disease definitions or normal ranges of blood pressure or lab values. We are not a consensus group on the BPD definition, nor are we attempting to collect data on reference values for lab disturbances. We used the available literature to provide the definitions and gradings as mentioned. If newer definitions or reference values become available, we will include them in a newer version.

Also important to know:

- We aimed to provide definitions for mild, moderate, severe and life-threatening forms of neonatal AEs, which can be used by researchers to standardize AE reporting. This is a consensus exercise, and a bit of pragmatism is required. A severity scale should be **easy to apply** by its users (research clinicians and nurses in different settings around the world), it should provide a **clear** cut-off between categories (decreasing interobserver variability), but on the other hand should also be **useable for any (atypical) form of a given AE** and thus be relatively open for interpretation. There should also be some **consistency** between the scales of different AE's and thus the specific gradings should be a clear reflection of the generic criteria.
- Where possible the **definitions of the Pediatric Adverse Event Terminology** (*Gipson et al, Pediatrics 2017*) were mentioned and the connected MedDRA term is referenced. If no existing definitions or terms were available, a proposition was worded. Attempts will be made to add these terms to MedDRA and PAET. For some there were question to alter the definition, we will ask this to the

governing bodies of this terminology. Attention should be drawn to the fact that a definition (can be pathophysiology or histology based) is not the same as diagnostic criteria.

Generic criteria

<table>
<tr><th>Grade 1</th><th>Grade 2</th><th>Grade 3</th><th>Grade 4</th><th>Grade 5</th></tr>
<tr><td>Mild</td><td>Moderate</td><td>Severe</td><td>Life threatening</td><td>Death</td></tr>
<tr><td>Mild; asymptomatic or mild symptoms; clinical or diagnostic observations only; no change in baseline age-appropriate behavior*; no change in baseline care or monitoring indicated</td><td>Moderate; resulting in minor changes of baseline age-appropriate behavior*; requiring minor changes in baseline care or monitoring***</td><td>Severe; resulting in major changes of baseline age-appropriate behavior* or non-life threatening changes in basal physiological processes**; requiring major change in baseline care or monitoring****</td><td>Life-threatening; Resulting in life-threatening changes in basal physiological processes**; requiring urgent major change in baseline care</td><td>Death related to AE</td></tr>
<tr><td colspan="5">*Age-appropriate behavior refers to oral feeding behavior, voluntary movements and activity, crying pattern, social interactions and perception of pain.
**Basal physiological processes refer to oxygenation, ventilation, tissue perfusion, metabolic stability and organ functioning.
***Minor care changes constitute: brief, local, non-invasive or symptomatic treatments
***Major care changes constitute: surgery, addition of long term treatment, upscaling care level</td></tr>
<tr><td colspan="5">If the different factors of this scale result in conflicting severity grades, the highest grade should be reported.</td></tr>
</table>

We remind you that long term outcome or prognosis is not a factor in the severity scale (as it is impossible to predict, or establish a causal link in individual cases) and should thus not be used in the specific scales either.

Specific criteria

Neurological

<table>
<tr><th>Grade 1</th><th>Grade 2</th><th>Grade 3</th><th>Grade 4</th><th>Grade 5</th></tr>
<tr><td>Mild</td><td>Moderate</td><td>Severe</td><td>Life threatening</td><td>Death</td></tr>
<tr><td colspan="5">Neonatal convulsion
Definition to be added|10010911: Sudden, involuntary, rapid, rhythmic or stereotyped skeletal muscular contractions in a newborn.</td></tr>
<tr><td>Single, self-limited suspected seizure</td><td>No recurrent suspected seizures within 3 days after treatment with 1 anti-seizure drug</td><td>Recurrence of suspected seizures within 3 days after treatment with 1 anti-seizure drug, or suspected seizures requiring 2 or more anti-seizure drugs</td><td>Suspected seizures with life-threatening consequences</td><td>Death related to suspected seizures</td></tr>
<tr><td colspan="5">25% disagreement (9/36)
Comments:
- Should suspected seizure be included separately from seizure?
- Any convulsion should be at least moderate as it requires at least increased observation (cfr. generic criteria)
- The difference between grade 3 and 4 ‘life threathening consequences’ should be clarified (e.g. supportive measures, status epilepticus) (x3)
- Wording of moderate is confusing (x4)</td></tr>
</table>

- The major difference between the categories is a treatment decision (might be variable among centers) (x4)<br>- Three or more drugs is a better cut-off, rather then 2 or more.<br>- Grade 1 might be difficult to capture in clinical reality (needs more explanation: desaturation, …)				
Single, self-limited suspected seizure, no treatment	Suspected seizures controlled with 1 anti-seizure drug (no recurrence within 3 days after treatment)	Suspected seizures uncontrolled with 1 anti-seizure drug (recurrence within 3 days after treatment or requiring 2 or more anti-seizure drugs)	Suspected seizures with life-threatening consequences (e.g. need for ventilation); suspected status epilepticus* despite multiple anti-seizure drugs	Death related to suspected seizures
*>30min duration of convulsions within any 60-minute period				
**Neonatal epileptic seizure**<br>Definition C2962 (to be added)|10039906 (to be added): *An EEG confirmed paroxysmal surge of electrical activity in the brain that may result in physical or behavioral changes in a neonate.*				
Single, self-limited seizure*	No recurrent seizures* within 3 days after treatment with 1 anti-seizure drug	Recurrence of seizures* within 3 days after treatment with 1 anti-seizure drug, or seizures* requiring 2 or more anti-seizure drugs	Status epilepticus* or seizures* with life-threatening consequences	Death related to seizures*
*Electrographic confirmation required: can be electro-clinical or electrographic only				
**26.5%** disagreement (9/34)<br>Comments:<br>- Difference between convulsion and seizure is not clear (x2), maybe add the need for EEG confirmation to the definition<br>- The difference between grade 3 and 4 'life threatening consequences' should be clarified (e.g. supportive measures)<br>- Should there be a distinction between focal and generalized seizures?<br>- Three or more drugs is a better cut-off, rather than 2 or more.<br>- Wording of moderate is confusing (x2)<br>- How is status epilepticus defined (duration?) (x3)?<br>- The major difference between the categories is a treatment decision (might be variable among centers)<br>- Epileptic seizures as seen on EEG should always be treated, so mild does not exist for this AE				
Single, self-limited EEG-proven seizure*, no treatment	EEG-proven seizures* controlled with 1 anti-seizure drug (no recurrence within 3 days after treatment)	EEG-proven seizures* uncontrolled with 1 anti-seizure drug (recurrence within 3 days after treatment or requiring 2 or more anti-seizure drugs)	EEG-proven seizures* with life-threatening consequences (e.g. need for ventilation); status epilepticus** despite multiple anti-seizure drugs	Death related to seizures*
*Electrographic confirmation required: can be electro-clinical or electrographic only<br>**>30min of seizure activity within any 60-minute period				
**Intraventricular hemorrhage** (IVH)<br>Definition C50896 (to be added)|10022841: *Bleeding into the lateral cerebral ventricles in a newborn infant.*				
Asymptomatic hemorrhage confined to the	Moderate hemorrhage occupying <50% of	Hemorrhage occupying >50% of the ventricle	Hemorrhage with parenchymal venous infarction;	Death

<table>
<tr><td>germinal matrix; minimal hemorrhage within the ventricle (<10% on parasagittal view)</td><td>ventricle volume (<50%) without ventricular dilatation > 4mm above the 97th percentile</td><td>volume; ventricular dilatation > 4mm above the 97th percentile; parenchymal venous infarction; requiring temporizing neurosurgical procedure (drain, shunt or reservoir)</td><td>resulting in life threatening consequences (e.g. refractory seizures, hypotension, respiratory depression, …); requiring urgent stabilization or surgical decompression</td><td></td></tr>
<tr><td colspan="5">16.1% disagreement (5/31)
Comments:
- I don’t agree with the definition: can lateral be omitted?
- The logical relationship between the criteria for grade 3 is unclear (and/or) (x4).
- Death is commonly due to withdrawal in IVH and thus subject of variable local practices.
- Respiratory depression is a bad discriminator as most neonates with high grade IVH are ventilated.
- Refractory seizures needs defining.
- Why are grade III and grade IV (venous parenchymal infarction) IVH taken together here as severe?
- The 50% limit is not reliable to measure. It also does not predict outcome, while ventricular dilatation does. Suggestion to omit the 50% limit and focus on dilatation.
- What about changing conditions after initial AE reporting?
- Does temporizing neurosurgical procedure mean planned, non-urgent intervention? Is this clear enough?
- Many neonatal neurologists would consider the Levene index to strict for a hydrocephalus definition?</td></tr>
<tr><td colspan="5">Retinopathy of prematurity (ROP)
Definition C34982|10038933: A retinal condition of very immature infants that may be characterized by non-vascularized retina that may lead to neovascularization, scarring, retinal detachment, and blindness.</td></tr>
<tr><td>Zone 2 ICROP stage 1 with or without plus disease; zone 2 ICROP stage 2 without plus disease; zone 3 any ICROP stage; no care changes indicated</td><td>Type 2 prethreshold ROP (zone 1 ICROP stage 1 or 2 without plus disease; zone 2 ICROP stage 3 without plus disease); requiring more frequent ophthalmic monitoring.</td><td>Type 1 prethreshold ROP (zone 1 any stage with plus disease; zone 1 ICROP stage 3 without plus disease; zone 2 ICROP stage 2 or 3 with plus disease); threshold ROP; requiring major care changes (e.g. laser intervention, intravitreal anti-VEGF or operative management)</td><td>Unilateral retinal detachment</td><td>Blindness (bilateral retinal detachment, …)</td></tr>
<tr><td colspan="5">10.3% disagreement (3/29)
Comments:
- Suggest to make unilateral retinal detachment grade 4 and bilateral retinal detachment grade 5, as death by ROP anyway does not exist.
- Zone 2 stage 2 without plus disease should be moved to moderate.
- Type 1 could also fall under moderate because of more frequent monitoring.</td></tr>
<tr><td colspan="5">Hypoxic Ischemic Encephalopathy (HIE)
Definition C35549|10070511: Injury to the central nervous system that occurs when there is insufficient delivery of oxygen to all or part of the brain.</td></tr>
</table>

<table>
<tr><td>Perinatal asphyxia event without signs of encephalopathy</td><td>Mild transient clinical signs of encephalopathy resulting from a perinatal asphyxia event, necessitating observation and additional care</td><td>Moderate clinical signs of encephalopathy resulting from a perinatal asphyxia event, necessitating therapeutic hypothermia</td><td>Severe clinical signs of encephalopathy resulting from a perinatal asphyxia event with life-threatening consequences</td><td>Death</td></tr>
<tr><td colspan="5">31.0% disagreement (9/29)
Comments:
- Mild: we should not classify it if there is no signs of encephalopathy. Perinatal asphyxia event is insufficient to define HIE. (x5)
We do feel it is important to provide a way to classify for instance notes in a medical record referring to asphyxia, or for instance the baby that was transferred after a bad start but did not display any signs of encephalopathy. This does not mean that it should be reported (can be defined in the protocol).
- Refer to Sarnat stages for mild-moderate-severe signs of encephalopathy. (x2)
- Severe signs of encephalopathy do not always correspond to life threathening consequences.
- Define additional care in moderate.
- Death is often due to withdrawal and thus subject to local practices.
- Refer to cooling criteria mentioned here http://hopefn3.org
- Severe should also include infants that do not meet the criteria for cooling (low birth weight, late presentation e.g.).</td></tr>
<tr><td>An event clinically classified as perinatal asphyxia, but not resulting in encephalopathy</td><td>Mild transient clinical signs* of encephalopathy resulting from a perinatal asphyxia event; necessitating observation and additional care</td><td>Moderate clinical signs* of encephalopathy resulting from a perinatal asphyxia event; meeting criteria for therapeutic hypothermia</td><td>Severe clinical signs* of encephalopathy resulting from a perinatal asphyxia event with life-threatening consequences (e.g. respiratory depression, refractory seizures)</td><td>Death</td></tr>
<tr><td colspan="5">*see Sarnat stages for further guidance on mild, moderate and severe clinical signs of encephalopathy</td></tr>
<tr><td colspan="5">Periventricular leukomalacia (PVL)
Definition C99013|10052594: A form of cerebral white matter injury usually seen in preterm infants that is characterized by necrotic degeneration or gliosis of white matter adjacent to the cerebral ventricles that may evolve into focal cysts.</td></tr>
<tr><td>Transient periventricular echo densities persisting > 7 days and resolving completely</td><td>Transient periventricular echo densities evolving in to small localized fronto-parietal cysts or persistent diffuse echodensities</td><td>Periventricular echo densities, evolving in to extensive cystic periventricular lesions; or densities extending in to the deep white matter</td><td>-</td><td>-</td></tr>
<tr><td colspan="5">14.3% disagreement (4/28)
Comments:
- This means two scans are needed at an interval of 7 days or just any time interval?
- Suggestion to add life-threatening and death (e.g. cystic lesions brainstem, decision to withdraw care because of extensive cystic PVL) (x3)
- Add echo densities </= 7days.
- Remove the localization specifier: can also evolve in to temporally located cysts.</td></tr>
</table>

**Infant irritability**<br>Definition C117267 | 10065283: *Crying easily, difficult to console.*				
Mild, self-limiting, irritability, not affecting feeding	Moderate irritability, minor changes in feeding behavior, requiring minor additional care	Severe irritability, major changes in feeding behavior, requiring support other than oral feeding; requiring medical treatment (e.g. opioids)	Life threatening irritability, with loss of autonomic control of temperature or heart rate; requiring urgent care change	-
**21.4%** disagreement (6/28)<br>Comments:<br>- Should we specify the context/cause of the irritability (e.g. drug withdrawal, noise, NAS, …) (x3)<br>- Life threatening irritability seems very unlikely. (x2)<br>- Add oxygenation or ventilation changes to life threatening<br>- Opioids seems a bit extreme as an example for medical treatment: add NSAIDs.<br>- Grade 3 may also include the need for additional diagnostic work-up.<br>- Why is only feeding included and no other parts of normal behavior (sleeping).<br>- Grade 1 has a very frequent occurrence (every day).<br>- Define urgent care changes.				
Mild, self-limiting, irritability, not affecting feeding and sleeping	Moderate irritability, minor changes in feeding and sleeping behavior, requiring minor additional care (e.g. occasional analgesics)	Severe irritability, major changes in feeding behavior, requiring support other than oral feeding; requiring long term medical treatment (e.g. sedatives)	Life threatening irritability, with loss of autonomic control of temperature or heart rate; requiring urgent care change	-
**Sedation**<br>Definition C118302 | 10039897: *A state of a lowered level of consciousness*				
Mild, self-limiting or transient sedation or sleepiness; transient loss of interactions; not requiring care change	Moderate sedation; limiting interactions and minor changes in feeding behavior; requiring increased monitoring	Severe sedation, major changes in feeding behavior; requiring support other than oral feeding (e.g. tube feeding)	Life-threatening sedation with cardiorespiratory instability; requiring urgent care change	-
**13.8%** disagreement (4/29)<br>Comments:<br>- The word sedation is unclear as it refers to analgesia or medication, while actually any cause of lowered consciousness should be considered. (x4)<br>- Maybe the need for stimulation could be added as an extra discriminator.<br>- Maybe respiratory depression could be used as an extra discriminator.<br>- There seems to be a major gap between grade 3 and 4.				

Cardiovascular

Grade 1	Grade 2	Grade 3	Grade 4	Grade 5
Mild	Moderate	Severe	Life threatening	Death
**Hypotension**<br>Definition C3128 | 10049223: *Abnormally low blood pressure, which is usually symptomatic.*				

-	Asymptomatic hypotension, not-affecting perfusion.	Persistent hypotension affecting perfusion, requiring major care change (e.g. vaso-active drugs or hydrocortisone).	Life-threatening consequences (e.g. shock, organ failure).	Death
**30.3%** disagreement (10/33)<br>Comments:<br>- BP norms are required. (x2)<br>- An asymptomatic AE, requiring no intervention should be mild. (x5)<br>- Consider adding 'volume bolus' as an additional discriminator (e.g. for moderate). (x3)<br>- Consider mild for transient (<60min) and moderate for persisting (>60/min).<br>- Consider using the need for additional diagnostic investigations as an additional discriminator (e.g for moderate).<br>- Define normal and affected perfusion and how it is measured. Consider altering "perfusion" to "organ perfusion". (x2)<br>- Definition of hypotension should be revisited.<br>- Add a possibility to attribute or indicate causality.<br>- Consider collapsing grade 4 and 5 as both would constitute a SAE.<br>- Based on treatment decisions and thus very variable among clinicians.				
Transient (<60/min), asymptomatic hypotension; not-affecting perfusion; not requiring intervention	Persistent (>60/min) hypotension; not affecting perfusion; requiring minor care changes (e.g. additional fluids)	Persistent hypotension affecting perfusion; requiring major care change (e.g. vaso-active drugs or hydrocortisone).	Life-threatening consequences (e.g. shock, organ failure).	Death
**Hypertension**<br>Definition C3117 | 10049781: *Abnormal high blood pressure.*				
Systolic or diastolic BP >90th percentile but <95th percentile; self-limiting.	Persistent or recurrent hypertension, with systolic or diastolic BP between the 95th percentile and the 99th percentile.	Persistent or recurrent hypertension, with systolic or diastolic above the 99th percentile; need for antihypertensive medication.	Life-threatening consequences (e. g. malignant hypertension: shock, cardiac failure or neonatal encephalopathy).	Death
For percentile values: e.g. *Dionne et al. Ped Nephrol 2012.*				
**12.1%** disagreement (4/33)<br>Comments:<br>- BP norms are required to make this usefull. (x3)<br>- Why still add a treatment decision for severe, if everything can be graded based on percentiles? Treatment decisions as discriminators introduce variability. (x2)<br>- Mean BP is probably a better parameter than systolic or diastolic<br>- Consider removing shock, but mentioning intracranial hemorrhage as this is the real risk.<br>- The diagnosis of hypertension requires several measurements, when the infant is calm: should this be specified?				
**Sinus tachycardia**<br>Definition C38029 | 10049775: *An abnormally high heart rate for age.*				
Brief, self-limiting, episodes of	Persistent tachycardia; no change in age-	Persistent tachycardia; resulting in non-	Life-threatening consequences; requiring urgent	Death

tachycardia; no care changes	appropriate behavior; requiring minor care changes (e.g. concomitant medication changed)	life threatening hemodynamic compromise; requiring major care changes (e.g. new medication or intervention)	major care changes	
**18.8%** disagreement (6/32)<br>Comments:<br>- What HR level is regarded as tachycardia? Consider using specific norms (for postnatal and gestational age).<br>- What does age appropriate behavior mean and is it a relevant discriminator here? (x2)<br>- Grade 1 seems too mild to report.<br>- Life threatening sinus tachycardia seems very unlikely to occur without underlying cause.				
**Sinus bradycardia**<br>Definition C37920|10056471: *An abnormally low heart rate for age.*				
Brief, self-limiting, episodes of bradycardia; no care changes	Persistent bradycardia; no change in age-appropriate behavior; requiring minor care changes (e.g. concomitant medication changed)	Persistent bradycardia; resulting in non-life threatening hemodynamic compromise; requiring major care changes (e.g. new medication or intervention)	Life-threatening consequences (e.g. shock); requiring urgent major care changes	Death
**21.2%** disagreement (7/33)<br>Comments:<br>- What HR level is regarded as bradycardia? Consider using specific norms (for postnatal and gestational age).<br>- Persistent bradycardia can be perfectly tolerated and should be classified as mild.<br>- What does age appropriate behavior mean and is it a relevant discriminator here? (x2)<br>- Death due to sinus bradycardia seems very unlikely to occur without underlying cause.<br>- How should grade 1 be distinguished from short self-resolving events related to apnea or GERD?<br>- Concomitant medication can be changed but also discontinued. Please specify this.<br>- Consider "adding oxygen" as an example of a minor care change.				
Brief, self-limiting, episodes of bradycardia; no care changes	Persistent bradycardia; no change in age-appropriate behavior; requiring minor care changes (e.g. concomitant medication adapted, intermittent increase FiO_2)	Persistent bradycardia; resulting in non-life threatening hemodynamic compromise; requiring major care changes (e.g. new medication or intervention)	Life-threatening consequences (e.g. shock); requiring urgent major care changes	Death
**Tachyarrhythmia**<br>Definition to be added|to be added (neonatal version of 10049447): *Non sinus rhythm with an abnormally high heart rate for age.*				
Brief, self-limiting, episodes of asymptomatic tachyarrhythmia (e.g. extrasystolic beats); no care changes	No change in age-appropriate behavior; requiring minor care changes (e.g. increased monitoring)	Resulting in non-life threatening hemodynamic compromise or changes in age-appropriate behavior; requiring	Life-threatening consequences (e.g. shock); requiring urgent major care changes	Death

<table>
<tr><td></td><td></td><td>major care changes (e.g. new medication or intervention)</td><td></td><td></td></tr>
<tr><td colspan="5">15.2% disagreement (5/33)
Comments:
- What does age appropriate behavior mean and is it a relevant discriminator here?
- Wording of moderate can be improved by starting with “episodes of tachyarrythmia”.
- Consider adding PAC as another example for mild.
- Consider adding SVT with decompensation, V tach, V fib as examples for life threatening.
- Any tachyarrithmia will probably require increased monitoring.
- I don’t think this should be classified as an adverse event as it mostly is the consequence of an underlying process (hyperkalemia e.g.).</td></tr>
<tr><td colspan="5">Bradyarrhythmia
Definition to be added|to be added (neonatal version of 10049765): Non sinus rhythm with an abnormally low heart rate for age.</td></tr>
<tr><td>Brief, self-limiting, episodes of asymptomatic bradyarrhythmia (e.g. extrasystolic beats); no care changes</td><td>No change in age-appropriate behavior; requiring minor care changes (e.g. increased monitoring)</td><td>Resulting in non-life threatening hemodynamic compromise; requiring major care changes (e.g. new medication or intervention)</td><td>Life-threatening consequences (e.g. shock); requiring urgent major care changes</td><td>Death</td></tr>
<tr><td colspan="5">30.3% disagreement (10/33)
Comments:
- What does age appropriate behavior mean and is it a relevant discriminator here?
- Perfusion could be used as a discriminator, but should be defined.
- The difference between grade 2 and grade 3 is not clear enough/these grades are the same. (x7) After revision we indeed noticed that there was an error in the survey duplicating the moderate criteria in the severe box.
- Very rare in newborns (AV-block). (x3)
- Extrasystolic beats would not lead to bradyarrythmia.
- This should not be considered as an adverse event.
- How can we distinguish this from sinus bradycardia, especially the mild forms?</td></tr>
<tr><td>Brief, self-limiting, episodes of asymptomatic bradyarrhythmia; no care changes</td><td>No change in age-appropriate behavior; requiring minor care changes (e.g. increased monitoring)</td><td>Resulting in non-life threatening hemodynamic compromise; requiring major care changes (e.g. new medication or intervention)</td><td>Life-threatening consequences (e.g. shock); requiring urgent major care changes</td><td>Death</td></tr>
<tr><td colspan="5">Edema
Definition C3002|10061317: Accumulation of an excessive amount of fluid in cells or intercellular tissues.</td></tr>
<tr><td>Mild edema; no change in age appropriate behavior; no care changes indicated</td><td>Moderate edema; no change in age appropriate behavior; requiring minor care changes (e.g. alteration in fluid management)</td><td>Severe edema; limiting age appropriate behavior; requiring major care changes (e.g. diuretics)</td><td>Life threatening consequences (e.g. respiratory failure, shock); requiring urgent major care changes (e.g. intubation, dialysis)</td><td>Death</td></tr>
<tr><td colspan="5">18.2% disagreement (6/33)
Comments:
- Edema needs to be defined better/more objectively. (x2)</td></tr>
</table>

- The decision to alter fluid management and to start diuretics is very clinician dependent. Suggestion to use fluid alterations or diuretics for moderate and fluid alterations AND diuretics for severe.<br>- Grade 4 and 5 are unlikely to be caused by edema only, without identified underlying cause.<br>- Diuretics are very common and should be a criterion for moderate instead of severe.				
**Coagulation disorder**<br>Definition C2902|10009732: *A condition of abnormal blood clotting or bleeding.*				
Minor biochemical coagulation abnormalities without clinical signs; no care changes indicated	Biochemical coagulation abnormalities with clinical signs; requiring increased monitoring	Biochemical or clinical coagulation abnormalities; requiring intervention	Life threatening consequences (e.g. severe pulmonary embolism, limb ischemia, hemorrhagic shock, DIC); requiring urgent major care changes	Death
**12.1%** disagreement (4/33)<br>Comments:<br>- References for coagulation times for gestational and postnatal age should be added here. (x2)<br>- Why would moderate just require increased monitoring?<br>- Does it matter if it is caused by an irreversible condition (e.g., liver failure) vs reversible (drug AE)?				

Respiratory

Grade 1	Grade 2	Grade 3	Grade 4	Grade 5
Mild	Moderate	Severe	Life threatening	Death
**Apnea**<br>Definition C26698|10077321: *Cessation of air flow.*				
Self-limiting apnea	Apnea requiring stimulation	Apnea requiring stimulation or FiO_2 increase; reoccurrences requiring respiratory stimulants or other major care changes	Life-threatening respiratory and/or hemodynamic compromise; ventilation required	Death
**34.4%** disagreement (11/32)<br>Comments:<br>- Self-limiting would need a duration specified.<br>- Change wording of moderate to "apnea responsive to stimulation".<br>- Include non-invasive ventilation as a discriminator for grade 3.<br>- Intubation or urgent ventilation should be the discriminator for grade 4. (x2)<br>- Suggestion to use an intermittent increase in FiO_2 as a discriminator for grade 2 (as this is done at a very low threshold).<br>- Apnea (especially mild) is a normal event in preterms and should not be reported as an AE. (x2)<br>- Suggestion to use stimulation AND FiO_2 increase as the discriminator for grade 3.<br>- The given definition is ambiguous, suggestion to specify length (20sec or 10 sec + desaturation or bradycardia). (x2)<br>- Suggestion to use frequency of the apneas as a discriminator: infrequent <3/day for moderate and frequent for severe.<br>- What about prophylactic caffeine?<br>- General comment: many of the language is not understandable for parents (important if we would envisage patient driven reporting).				
Self-limiting apnea	Apnea responsive to stimulation or	Apnea requiring stimulation and sustained FiO_2	Life-threatening respiratory and/or hemodynamic	Death

	intermittent FiO_2-increase.	increase; requiring non-invasive ventilation; reoccurrences requiring start of or relevant increase in dose of respiratory stimulants or other major care changes	compromise; (semi-)urgent intubation required	
**Respiratory insufficiency**<br>Definition to be added│to be added: *The significant impairment of gas exchange resulting in compensatory breathing efforts and eventually hypoxia and/or hypercarbia.**				
-	Clinical deterioration of respiratory distress without relevant increase in PCO_2 or decrease in oxygenation	Clinical deterioration of respiratory distress; with relevant increase in PCO_2 or decrease in oxygenation, requiring major care change (e.g. start of non-invasive ventilatory support, adaptation of current ventilatory support mode)	Life-threatening respiratory and/or hemodynamic compromise; requiring urgent care change (e.g. intubation, start of invasive ventilatory support)	Death
**Consider changes from baseline condition*				
**25.8%** disagreement (8/31)<br>Comments:<br>- There seems to be a lot of overlap between grade 3 and 4.<br>- What about preterm babies that are often intubated already before they develop respiratory distress or hemodynamic compromise. This is not always life threatening. (x2)<br>- Include increased work of breathing as a criterion for mild.<br>- The current wording “clinical deterioration of respiratory distress” is confusing (e.g. use status, use clinical evidence). (x2)<br>- Suggestion to also include a mild category. (x3)<br>- Suggestion to use ‘increase FiO_2 without positive pressure’ as a marker for moderate. (x3)<br>- Severe should be more severe.<br>- How can we identify increased respiratory distress without alterations in gas exchange?				
Clinical evidence of mildly increased respiratory distress (e.g. increased work of breathing) without significant deterioration in gas exchange (increase in pCO2 or decrease in oxygenation); no change in baseline functioning; no care changes required	Clinical evidence of increased respiratory distress without significant deterioration in gas exchange (increase in pCO2 or decrease in oxygenation); corrected by minor adjustments in current ventilatory support, supplemental oxygen or non-invasive support	Clinical evidence of increased respiratory distress with relevant deterioration in gas exchange (increase in pCO2 or decrease in oxygenation); requiring major care change (e.g. start invasive support)	Life-threatening respiratory and/or hemodynamic compromise; requiring urgent care change (e.g. urgent intubation)	Death

<table>
<tr><td colspan="5">*Consider this adverse event for any patient whose respiratory condition deteriorates from baseline. If any specific diagnosis is identified: use diagnosis specific scale (e.g. RDS, pulmonary hemorrhage)</td></tr>
<tr><td colspan="5">Respiratory distress syndrome (RDS)
Definition C27560|10028974: Progressive alveolar atelectasis from birth due to an abnormality of synthesis, function or metabolism of surfactant, characterized by respiratory failure and an abnormal chest radiograph showing diffuse reticulogranular densities and air bronchograms.</td></tr>
<tr><td>Radiological evidence without clinical signs</td><td>Clinically increased respiratory distress, without relevant increase in PCO_2 or decrease in oxygenation, requiring non-invasive respiratory support for <24h</td><td>Clinically increased respiratory distress, with relevant increase in PCO_2 or decrease in oxygenation, requiring non-invasive respiratory support for >24h or other major care change</td><td>Life-threatening respiratory and/or hemodynamic compromise; requiring urgent intervention or mechanical ventilation</td><td>Death</td></tr>
<tr><td colspan="5">22.6% disagreement (7/31)
Comments:
- Should RDS be listed as a neonatal adverse event?
- Should the need for surfactant be included as a severity criterion?
- Non-invasive ventilation could be specified more (nasal canula, CPAP, HFNV)
- Why does this vary from respiratory insufficiency? Do we need both?
- Severe should be more severe. (x2)
- The current mild definition is too mild to report.
- Suggestion to keep <24h non-invasive support for mild and longer periods for non-invasive support for moderate.
- Suggestion to keep any duration of non-invasive support for moderate.
- Suggestion to make invasive support severe. (x2)</td></tr>
<tr><td>Radiological evidence without clinical signs; clinical evidence of mildly increased respiratory distress (e.g. increased work of breathing) without significant deterioration in gas exchange (increase in pCO2 or decrease in oxygenation); no supportive care required</td><td>Clinical evidence of increased respiratory distress without significant deterioration in gas exchange (increase in pCO2 or decrease in oxygenation); corrected by minor adjustments in current ventilatory support, supplemental oxygen or non-invasive support</td><td>Clinical evidence of increased respiratory distress with relevant deterioration in gas exchange (increase in pCO2 or decrease in oxygenation); requiring major care change (e.g. start invasive support)</td><td>Life-threatening respiratory and/or hemodynamic compromise; requiring urgent care change (e.g. urgent intubation)</td><td>Death</td></tr>
<tr><td colspan="5">Pulmonary hemorrhage
Definition to be added|10038728: Bleeding in the respiratory tract of a neonate.</td></tr>
<tr><td>Limited hemorrhagic secretion in ET tube</td><td>Hemorrhagic secretion in ET tube; without relevant increase in PCO_2 or decrease in oxygenation; requiring minor changes in care (e.g. increase PEEP)</td><td>Hemorrhagic secretion in ET tube; with relevant increase in PCO_2 or decrease in oxygenation; requiring major change in ventilatory support or transfusion</td><td>Life-threatening respiratory and/or hemodynamic compromise</td><td>Death</td></tr>
</table>

**10%** disagreement (3/30)<br>Comments:<br>- Increasing PEEP often helps solving this problem: maybe resolving after increasing PEEP versus refractory to increased PEEP could be another discriminator.<br>- Should we not discriminate between blood from upper respiratory tract (e.g. from suctioning) versus true pulmonary hemorrhage.<br>- Hemorrhagic pulmonary edema would be a better term.<br>- Grade 1 is too mild to report.<br>- What levels of PCO_2 and oxygenation are relevant?				
**Persistent pulmonary hypertension of the newborn** (PPHN)<br>Definition to be added | to be added: *Elevated pulmonary vascular pressure in a neonate.*				
Technical evidence of increased RV-pressures; no clinical symptoms	Increased estimated RV-pressures; with moderate clinical symptoms; oxygenation index <25; minor care changes required	Increased estimated RV-pressures; with severe clinical symptoms; oxygenation index >25; major care changes required (e.g. iNO)	Life-threatening respiratory and/or hemodynamic compromise; ECMO required; oxygenation index >40	Death
**17.2%** disagreement (5/29)<br>Comments:<br>- RV-pressure cut-offs are needed for grading.<br>- Severe for this diagnosis feels much more severe, than severe for other AEs.<br>- There is not much discrimination between severe and life-threatening.<br>- OI does not always guide clinical practice.<br>- Add OI>4 for life-threatening. (x2)<br>- Respiratory support modes could be considered.<br>- The OI limits are too severe: suggestion to use 10-15, 15-20 and 20-25.<br>- Suggestion to distinguish postnatal pulmonary hypertension (PPHN) from longer term PH related to CLD, …				
**Pneumothorax**<br>Definition C38006 | to be added: *A collection of air or other gas between the visceral and parietal pleura.*				
Radiological evidence of pneumothorax; no clinical signs; no care change required	Radiological evidence of pneumothorax; minor clinical signs; minor care change required (e.g. oxygen, increased monitoring)	Radiological evidence of pneumothorax; with clinical signs; major care change required (e.g. chest drainage)	Life-threatening respiratory and/or hemodynamic compromise (e.g. tension pneumothorax); urgent major care change required	Death
**13.8%** disagreement (4/29)<br>Comments:<br>- Should single needle aspiration be included as an example of a minor/major care change? (x2)<br>- Suggestion to add bronchopleural fistula to e.g.<br>- Ultrasound or translumination could also be valid diagnostic techniques.				
**Bronchopulmonary dysplasia**<br>Definition C90599|10006475: *A chronic lung disorder associated with pulmonary maldevelopment, scarring, and/or inflammation that develops in preterm neonates. The condition is defined based on treatment with supplemental oxygen for at least 28 days adjusted for the degree of prematurity.*				
Breathing room air at 36 weeks PMA in infants born <32 weeks' gestation; breathing room air by 56 days	Need for <30% oxygen at 36 weeks PMA in infants born <32 weeks' gestation; need for <30% oxygen by 56	Need for >30% oxygen and/or positive pressure at 36 weeks PMA in infants born <32 weeks' gestation;	-	-

<table>
<tr><td>postnatal age in infants born >32 weeks’ gestation; breathing room air at discharge</td><td>days postnatal age in infants born >32 weeks’ gestation; need for <30% oxygen at discharge</td><td>need for >30% oxygen and/or positive pressure by 56 days postnatal age in infants born >32 weeks’ gestation; need for >30% oxygen and/or positive pressure at discharge</td><td></td><td></td></tr>
<tr><td colspan="5">37.9% disagreement (11/29)
Comments:
- The diagnosis of BPD has changed importantly since 2001, please use a more up to date reference or use a newer consensus definition. (x3)
- Oxygen requirement is not a good criterion, as many centers do not perform oxygen tests.
- Oxygen requirement is not a good criterion, as many centers use different saturation targets.
- Oxygen requirement in FiO_2 is not a good criterion, as most oxygen is administered as l/min by nasal canula.
- The place to which the patient is discharged (home, step down center) should be specified.
- The definition should be more clear (include ‘initial’).
- Grade 4 should be added (e.g. longer than 6 months, affecting life style, tracheostomy, home ventilation). (x5)
- Grade 5 is possible as well.
- It looks like every baby should be mild BPD this way? Please make this clearer. (x2)
- Write 22-29% instead of <30%.</td></tr>
<tr><td>Supplemental oxygen at 28 days AND
Breathing room air at 36 weeks PMA in infants born <32 weeks’ gestation; breathing room air by 56 days postnatal age in infants born >32 weeks’ gestation; breathing room air at discharge</td><td>Supplemental oxygen at 28 days AND
Need for 22-30% oxygen at 36 weeks PMA in infants born <32 weeks’ gestation; need for 22-30% oxygen by 56 days postnatal age in infants born >32 weeks’ gestation; need for 22-30% oxygen at discharge</td><td>Supplemental oxygen at 28 days AND
Need for >30% oxygen or positive pressure at 36 weeks PMA in infants born <32 weeks’ gestation; need for >30% oxygen or positive pressure by 56 days postnatal age in infants born >32 weeks’ gestation; need for >30% oxygen and/or positive pressure at discharge</td><td>Supplemental oxygen at 28 days AND
Need for >30% oxygen AND positive pressure at 36 weeks PMA in infants born <32 weeks’ gestation; need for >30% oxygen AND positive pressure by 56 days postnatal age in infants born >32 weeks’ gestation; need for >30% oxygen AND positive pressure at discharge</td><td>Death</td></tr>
<tr><td colspan="5">For conversion of oxygen administered by different modalities to FiO_2: see conversion tables</td></tr>
</table>

Gastro-intestinal

<table>
<tr><th>Grade 1</th><th>Grade 2</th><th>Grade 3</th><th>Grade 4</th><th>Grade 5</th></tr>
<tr><td>Mild</td><td>Moderate</td><td>Severe</td><td>Life threatening</td><td>Death</td></tr>
<tr><td colspan="5">Necrotising enterocolitis (NEC)
Definition C84915|10055667: A disease of neonates in which there is extensive mucosal ulceration, pseudomembrane formation, submucosal hemorrhage, and necrosis.</td></tr>
</table>

-	-	NEC confirmed; major care change indicated (e.g. NPO, antibiotics, non-urgent surgery)	Bowel perforation (pneumoperitoneum) (Bell IIIB); shock, DIC, combined respiratory and metabolic acidosis (Bell IIIA); urgent major care change indicated	Death
If NEC is not confirmed (Bell stages I): please record severity of individual symptoms (e.g. feeding intolerance).				
**22.2%** disagreement (6/27)<br>Comments:<br>- Suggestion to add mild and moderate (e.g. abdominal distension or feeding intolerance). (x3)<br>- Good idea to keep abdominal distension and feeding intolerance out.<br>- Do we need radiological confirmation (pneumatosis) or is a clinical diagnosis enough?. (x2)<br>- A moderate form of NEC might still exist for which NPO and antibiotics are started (e.g. a few flecks for pneumatosis, <25%).<br>- The definition is completely based on pathology and is thus not clinically usable.<br>- Moderate and severe could be used to distinguish NEC needing antibiotics versus NEC needing surgery. (x2)				
-	-	NEC confirmed; major care change indicated (e.g. NPO, antibiotics, non-urgent surgery)	Bowel perforation (pneumoperitoneum) (Bell IIIB); shock, DIC, combined respiratory and metabolic acidosis (Bell IIIA); urgent major care change indicated	Death
If NEC is not confirmed (Bell stages I): please record severity of individual symptoms (e.g. feeding intolerance).				
**Diarrhea**<br>Definition C2987 | 10012743: *Watery bowel movements.*				
Increase of 2-4 stools per day over baseline; mild increase in ostomy output compared to baseline	Increase of 4 - 6 stools per day over baseline; moderate increase in ostomy output compared to baseline	Increase of >=7 stools per day over baseline; severe increase in ostomy output compared to baseline; signs of dehydration	Life-threatening consequences (e.g. severe dehydration)	Death
**7.6%** disagreement (2/27)<br>Comments:<br>- Volume could be more accurate (ml/kg) than frequency<br>- Should baseline be specified as the average stool frequency of the last 3?? days?<br>- Baseline stool frequency will automatically be very variable in newborns (meconium e.g.), should baseline be further specified?<br>- Mild and moderate ostomy output could be better delineated (%?)?				
**Vomiting**<br>Definition C3442 | 10075315: *Expulsion of the contents of the stomach through the mouth.*				
Increase in vomiting over baseline, self-limiting.	Persistent increase in vomiting over baseline; no dehydration; minor changes in feeding support indicated	Persistent increase in vomiting over baseline; signs of dehydration; major changes in feeding support indicated (e.g.	Life-threatening consequences (e. g. severe dehydration)	Death

<table>
<tr><td></td><td></td><td>change to TPN or gavage)</td><td></td><td></td></tr>
<tr><td colspan="5">7.6% disagreement (2/27)
Comments:
- Not sure if gavage tube should be considered severe.
- Not sure if over baseline is helpful here.
- Define persistent.</td></tr>
<tr><td colspan="5">Feeding intolerance
Definition C113395 | 10076042: Inability to achieve a full feeding volume.</td></tr>
<tr><td>Mild feeding intolerance (e.g. increased gastric residue or abdominal distension); without discomfort; no change in care indicated</td><td>Moderate feeding intolerance; resulting in minor discomfort or alteration of drinking behavior; minor care changes indicated</td><td>Severe feeding intolerance; requiring major changes in feeding support indicated (e.g. change to TPN or gavage)</td><td>Life-threatening consequences</td><td>Death</td></tr>
<tr><td colspan="5">50% disagreement (13/26)
Comments:
- Mild and moderate appear to be the same. (x12)
After revision there was indeed an error in this question of the survey explaining the high disagreement. This item has thus been kept unchanged.
- Residual volume is better English than residue.
- Gavage feeding is a standard feeding for preterm infants.
- Life threatening feeding intolerance seems very unlikely without underlying condition specified.
- Volume of residuals could be quantified.
- Checking gastric residual volumes is not recommended in routine clinical care.</td></tr>
<tr><td>Mild feeding intolerance (e.g. increased gastric residual volume or abdominal distension); without discomfort; no change in care indicated</td><td>Moderate feeding intolerance; resulting in minor discomfort or alteration of drinking behavior; minor care changes indicated (e.g. feeds withheld)</td><td>Severe feeding intolerance; requiring major changes in feeding support indicated (e.g. change to TPN or gavage)</td><td>Life-threatening consequences</td><td>Death</td></tr>
<tr><td colspan="5">Gastro-intestinal bleeding
Definition C48592 | 10017955: Hemorrhage originating at any site located within the gastrointestinal tract.</td></tr>
<tr><td>Mild, self-limiting, bleeding; no change in care required</td><td>Moderate bleeding; minor change in care or monitoring required</td><td>Severe bleeding; non-life threatening hemodynamic consequences; major care change required (e.g. invasive intervention, transfusion, long term medical treatment)</td><td>Life-threatening consequences (e.g.hemorrhagic shock); urgent major care change required</td><td>Death</td></tr>
<tr><td colspan="5">7.7% disagreement (2/26)
Comments:
- Please provide examples of short term treatment (short term anti-acids, feeding withheld) (x2)</td></tr>
</table>

- Intermittently detected blood in stool (positive test) could be added for mild.<br>- Is it possible to have some bleeding that does not require additional monitoring?				
**Spontaneous intestinal perforation** (SIP)<br>Definition to be added | 10074160: *A perforation in the gastrointestinal tract of a newborn with no demonstrable cause.*				
-	-	Presence of SIP, non-urgent medical stabilization and surgical intervention indicated	Life-threatening consequences (e.g. shock, organ failure); urgent intervention indicated	Death
**3.7%** disagreement (1/27)<br>Comments:<br>- Mild and moderate are needed here as well.				
**Constipation**<br>Definition C37930 | 10010774: *Irregular and infrequent or difficult evacuation of the bowels.*				
Reduced number of stools or hard stools; no apparent discomfort	Reduced number of stools or hard stools; apparent discomfort; minor care changes indicated	Reduced number of stools or hard stools; signs of obstruction; apparent discomfort or affecting feeding; major care changes indicated (e.g. long term medical treatment, enema)	Life-threatening consequences	Death
**7.4%** disagreement (2/27)<br>Comments:<br>- Is number of stools a relevant parameter in neonates? (x2)<br>- Add the need for a diagnostic work-up (RX, …) as a severity discriminator<br>- Grade 1 is too mild to report				

Infectious

Grade 1	Grade 2	Grade 3	Grade 4	Grade 5
Mild	Moderate	Severe	Life threatening	Death
**Culture negative sepsis**<br>Definition to be added | 10040049 (to be added): *A systemic inflammatory response without identifiable cause.*				
-	Suspected sepsis with mild or ambiguous signs; anti-infectives initiated	Suspected sepsis with severe signs (e.g. fever, grunting); support treatment escalated or initiated	Life-threatening consequences (e.g. state of shock, DIC); urgent major care change required	Death
**0%** disagreement (0/24)<br>Comments:<br>- Please highlight the 'unidentifiable cause'<br>- Suggestion to use an inflammatory marker like CRP as a cut-off<br>- Add poor perfusion, change of tone to the clinical signs				
**Culture positive sepsis**				

Definition to be added|10040049 (to be added): *A systemic inflammatory response to an infection.*				
Blood culture positive; no care change indicated (e.g. contamination suspected)	Blood culture positive with mild or ambiguous signs; anti-infectives initiated	Blood culture positive with severe signs; support treatment escalated or initiated	Life-threatening consequences (e. g. state of shock, DIC); urgent major care change required	Death
**16.7%** disagreement (4/24)<br>Comments:<br>- Suggestion to use an inflammatory marker like CRP as a cut-off<br>- Suggestion to use the need for lumbar puncture or CSF infection as a discriminator (x2)<br>- Not sure if we should support not treating a positive blood culture, usually it is just stopped early. Usually you don't know whether it is a contaminant after a few days of treatment (x3)<br>- Why would a contaminant be an AE? (x2)<br>- Not sure if mild can be considered as a sepsis<br>- Suggestion to expand to topical cultures				

General

Grade 1	Grade 2	Grade 3	Grade 4	Grade 5
Mild	Moderate	Severe	Life threatening	Death
**Rash**<br>Definition: C39594|10037871: *An eruption in the skin which affects its appearance and/or texture.*				
Localized rash	Diffuse rash or target lesions	Diffuse rash and vesicles or limited number of bullae or superficial ulcerations of mucous membrane limited to one site	Extensive or generalized bullous lesions or ulceration of mucous membrane involving 2 or more distinct mucosal sites or stevens-johnson syndrome or toxic epidermal necrosis	Death
**5.7%** disagreement (2/35)<br>Comments:<br>- Would a limited number of mucous membrane ulcerations not be considered as SJS or TEN?				
**Administration site complication**<br>Definition to be added|to be added: *Local irritation or complication at the administration site of a drug.*				
Painless edema	Erythema with associated symptoms (e.g., edema, pain, induration, phlebitis)	Ulceration or necrosis; severe tissue damage; operative intervention indicated	Life-threatening consequences; urgent intervention indicated	Death
**14.3%** disagreement (5/35)<br>Comments:<br>- How is painless defined in a neonate?<br>- Hyaluronidase injections should be added here as well, as a marker for moderate. (x2)<br>- Anti-inflammatory treatment (NSAID or other) should be included as well, as a marker for moderate and severe.<br>- Specify whether this means by IV or other administration method<br>- Grade 1 should be redness + swelling.				
**Fever**				

Definition C3038|10016562: *Elevation of body temperature above normal due to the production of more heat than the body is able to dissipate.*				
Isolated, not sustained fever, no change in baseline age-appropriate behavior	Fever, minor change in baseline age-appropriate behavior; minor changes in care (e.g. environmental settings, symptomatic treatment)	Fever, major change in baseline age-appropriate behavior and without resolution to symptomatic treatment.	Life-threatening consequences	Death
**14.3%** disagreement (5/35)<br>Comments:<br>- Consider hypothermia and temperature instability as well.<br>- Suggestion to address environmental etiology of fever.<br>- Suggestion to address iatrogenic nature of fever.<br>- Fever is not a diagnosis, it is a sign, and should hence not be regarded as an AE.<br>- Define sustained.<br>- Define temperature limit for the diagnosis for fever, and for the different severity grades. (x2)<br>- Does symptomatic treatment include antipyretics?				
