# Supplementary material for: Development of a neonatal adverse event severity scale through a Delphi consensus approach
Source: Arch Dis Child. 2019 Sep 19;104(12):1167–73. doi: 10.1136/archdischild-2019-317399 (PMC6943241; doi:10.1136/archdischild-2019-317399)
Supplement: Supplementary data [file archdischild-2019-317399supp006.pdf]

Appendix 6

INC Neonatal Adverse Event Scale v1.0

Introduction

The INC Neonatal Adverse Event Severity Scale is a terminology that can be used to report adverse events (AEs). This document provides a descriptive severity scale for the most common neonatal AEs[1]. This document has been developed within INC[2].

An **AE** is defined as any untoward medical occurrence in a patient or clinical investigation subject administered a pharmaceutical product and which does not necessarily have a causal relationship with this treatment[3]. An AE is a term that is a unique representation of a specific event used for medical documentation and scientific analyses.

MedDRA and other terminology

All AE terms in this document have been linked to lowest level terms (LLT) in MedDRA (from v22.0 on). Definitions for adverse events where, where possible based on the NCI Thesaurus (NCIt) Pediatric Adverse Event Terminology Subset[4]. The brief descriptive definition is given for every AE term, to clarify its meaning. These are not diagnostic criteria. The National Cancer Institute (NCI) Enterprise Vocabulary Services (EVS) and INC have collaborated in this effort and EVS will house and maintain terminology to support the efforts of INC.

In this document all AE's are linked to the corresponding *EVS-code* | *MedDRA-code*.

Severity grades

All AE severity grades follow this generic framework. If there are no severity scales specified for an AE, this generic scale should be used.

<table>
<tr><th>Grade 1</th><th>Grade 2</th><th>Grade 3</th><th>Grade 4</th><th>Grade 5</th></tr>
<tr><td>Mild</td><td>Moderate</td><td>Severe</td><td>Life threatening</td><td>Death</td></tr>
<tr><td>Mild; asymptomatic or mild symptoms; clinical or diagnostic observations only; no change in baseline age-appropriate behavior*; no change in baseline care or monitoring indicated</td><td>Moderate; resulting in minor changes of baseline age-appropriate behavior*; requiring minor changes in baseline care or monitoring***</td><td>Severe; resulting in major changes of baseline age-appropriate behavior* or non-life threatening changes in basal physiological processes**; requiring major change in baseline care or monitoring****</td><td>Life-threatening; Resulting in life-threatening changes in basal physiological processes**; requiring urgent major change in baseline care</td><td>Death related to AE</td></tr>
<tr><td colspan="5">*Age-appropriate behavior refers to oral feeding behavior, voluntary movements and activity, crying pattern, social interactions and perception of pain.
**Basal physiological processes refer to oxygenation, ventilation, tissue perfusion, metabolic stability and organ functioning.
***Minor care changes constitute: brief, local, non-invasive or symptomatic treatments
***Major care changes constitute: surgery, addition of long term treatment, upscaling care level</td></tr>
<tr><td colspan="5">If the different factors of this scale result in conflicting severity grades, the highest grade should be reported.</td></tr>
</table>

A single dash (-) indicates a severity grade is not available. Not all grades are appropriate for all AEs.

Salaets T, *et al. Arch Dis Child* 2019; 104:1167–1173. doi: 10.1136/archdischild-2019-317399

1. *A priority list for AEs was determined with a survey among neonatologists and employees of industry and regulatory authorities. No lab values were included in this first version because of the lack of reference values for neonates. Any AE that is not listed can be graded according to the generic criteria.*
2. *https://c-path.org/programs/inc/*
3. *http://www.ich.org/products/guidelines/efficacy/efficacy-single/article/clinical-safety-data-management-definitions-and-standards-for-expedited-reporting.html*
4. *Gipson DS, Kirkendall ES, Gumbs-Petty B, Quinn T, Steen A, Hicks A, et al. Development of a Pediatric Adverse Events Terminology. Pediatrics. 2017;139(1).*

Work in progress

We anticipate this document will mature further with use and user feedback. Requests to add a new AE or to adapt some of the scales in this document can be filed through: https://ncitermform.nci.nih.gov/ncitermform/?dictionary=NCI%20Thesaurus

Neurological

Grade 1	Grade 2	Grade 3	Grade 4	Grade 5
Mild	Moderate	Severe	Life threatening	Death
**Neonatal Convulsion**<br>Definition C154952 | 10028932: *Sudden, involuntary, rapid, rhythmic or stereotyped skeletal muscular contractions in a newborn.*				
Single, self-limited suspected seizure, no treatment	Suspected seizures controlled with 1 anti-seizure drug (no recurrence within 3 days after treatment)	Suspected seizures uncontrolled with 1 anti-seizure drug (recurrence within 3 days after treatment or requiring 2 or more anti-seizure drugs)	Suspected seizures with life-threatening consequences (e.g. need for ventilation); suspected status epilepticus* despite multiple anti-seizure drugs	Death related to suspected seizures
*>30min duration of convulsions within any 60-minute period				
**Neonatal Epileptic Seizure**<br>Definition C154934 | 10082068 (v22.0): *An EEG confirmed paroxysmal surge of electrical activity in the brain that may result in physical or behavioral changes in a neonate.*				
Single, self-limited EEG-proven seizure*, no treatment	EEG-proven seizures* controlled with 1 anti-seizure drug (no recurrence within 3 days after treatment)	EEG-proven seizures* uncontrolled with 1 anti-seizure drug (recurrence within 3 days after treatment or requiring 2 or more anti-seizure drugs)	EEG-proven seizures* with life-threatening consequences (e.g. need for ventilation); status epilepticus** despite multiple anti-seizure drugs	Death related to seizures*
*Electrographic confirmation required: can be electro-clinical or electrographic only<br>**>30min of seizure activity within any 60-minute period				
**Neonatal Intraventricular Hemorrhage** (IVH)<br>Definition C154937 | 10022844: *Bleeding into the lateral cerebral ventricles in a newborn infant.*				
Asymptomatic hemorrhage confined to the germinal matrix; minimal hemorrhage within the ventricle (<10%	Moderate hemorrhage occupying <50% of ventricle volume (<50%) without ventricular dilatation > 4mm	Hemorrhage occupying >50% of the ventricle volume; ventricular dilatation > 4mm above the 97th percentile; parenchymal	Hemorrhage with parenchymal venous infarction; resulting in life threatening consequences (e.g. refractory seizures, hypotension,	Death

Salaets T, *et al. Arch Dis Child* 2019; 104:1167–1173. doi: 10.1136/archdischild-2019-317399

on parasagittal view)	above the 97th percentile	venous infarction; requiring temporizing neurosurgical procedure (drain, shunt or reservoir)	respiratory depression, …); requiring urgent stabilization or surgical decompression	
**Retinopathy of Prematurity** (ROP)<br>Definition C154925 │ 10038933: *A retinal condition of very immature infants that may be characterized by non-vascularized retina that may lead to neovascularization, scarring, retinal detachment, and blindness.*				
Zone 2 ICROP stage 1 with or without plus disease; zone 2 ICROP stage 2 without plus disease; zone 3 any ICROP stage; no care changes indicated	Type 2 prethreshold ROP (zone 1 ICROP stage 1 or 2 without plus disease; zone 2 ICROP stage 3 without plus disease); requiring more frequent ophthalmic monitoring.	Type 1 prethreshold ROP (zone 1 any stage with plus disease; zone 1 ICROP stage 3 without plus disease; zone 2 ICROP stage 2 or 3 with plus disease); threshold ROP; requiring major care changes (e.g. laser intervention, intravitreal anti-VEGF or operative management)	Unilateral retinal detachment	Blindness (bilateral retinal detachment, …)
**Hypoxic Ischemic Encephalopathy** (HIE)<br>Definition C154945 │ 10070512: *Injury to the central nervous system that occurs when there is insufficient delivery of oxygen to all or part of the brain.*				
An event clinically classified as perinatal asphyxia, but not resulting in encephalopathy	Mild transient clinical signs* of encephalopathy resulting from a perinatal asphyxia event; necessitating observation and additional care	Moderate clinical signs* of encephalopathy resulting from a perinatal asphyxia event; meeting criteria for therapeutic hypothermia	Severe clinical signs* of encephalopathy resulting from a perinatal asphyxia event with life-threatening consequences (e.g. respiratory depression, refractory seizures)	Death
*see Sarnat stages for further guidance on mild, moderate and severe clinical signs of encephalopathy				
**Periventricular Leukomalacia** (PVL)<br>Definition C154923 │ 10052594*: A form of cerebral white matter injury usually seen in preterm infants that is characterized by necrotic degeneration or gliosis of white matter adjacent to the cerebral ventricles that may evolve into focal cysts.*				
Transient periventricular echo densities persisting > 7 days and resolving completely	Transient periventricular echo densities evolving in to small localized fronto-parietal cysts or persistent diffuse echodensities	Periventricular echo densities, evolving in to extensive cystic periventricular lesions; or densities extending in to the deep white matter	-	-
**Infant Irritability**<br>Definition C154939 │ 10082189 (v22.0): *Crying easily, difficult to console.*				

Mild, self-limiting, irritability, not affecting feeding and sleeping	Moderate irritability, minor changes in feeding and sleeping behavior, requiring minor additional care (e.g. occasional analgesics)	Severe irritability, major changes in feeding behavior, requiring support other than oral feeding; requiring long term medical treatment (e.g. sedatives)	Life threatening irritability, with loss of autonomic control of temperature or heart rate; requiring urgent care change	-
**Infant Sedation**<br>Definition C154926 | 10082187 (v22.0): *A state of a lowered level of consciousness*				
Mild, self-limiting or transient sedation or sleepiness; transient loss of interactions; not requiring care change	Moderate sedation; limiting interactions and minor changes in feeding behavior; requiring increased monitoring	Severe sedation, major changes in feeding behavior; requiring support other than oral feeding (e.g. tube feeding)	Life-threatening sedation with cardiorespiratory instability; requiring urgent care change	-

Cardiovascular

Grade 1	Grade 2	Grade 3	Grade 4	Grade 5
Mild	Moderate	Severe	Life threatening	Death
**Neonatal Hypotension**<br>Definition C154930 | 10049223: *Abnormally low blood pressure, which is usually symptomatic.*				
Transient (<60/min), asymptomatic hypotension; not-affecting perfusion; not requiring intervention	Persistent (>60/min) hypotension; not affecting perfusion; requiring minor care changes (e.g. additional fluids)	Persistent hypotension affecting perfusion; requiring major care change (e.g. vaso-active drugs or hydrocortisone).	Life-threatening consequences (e.g. shock, organ failure).	Death
**Neonatal Hypertension**<br>Definition C154929 | 10049781: *Abnormal high blood pressure.*				
Systolic or diastolic BP >90th percentile but <95th percentile; self-limiting.	Persistent or recurrent hypertension, with systolic or diastolic BP between the 95th percentile and the 99th percentile.	Persistent or recurrent hypertension, with systolic or diastolic above the 99th percentile; need for antihypertensive medication.	Life-threatening consequences (e. g. malignant hypertension: shock, cardiac failure or neonatal encephalopathy).	Death
For percentile values: e.g. *Dionne et al. Ped Nephrol 2012.*				
**Neonatal Sinus Tachycardia**<br>Definition C154932 | 10082191 (v22.0): *An abnormally high heart rate for age.*				
Brief, self-limiting, episodes of tachycardia; no care changes	Persistent tachycardia; no change in age-appropriate behavior; requiring minor care changes	Persistent tachycardia; resulting in non-life threatening hemodynamic compromise; requiring major	Life-threatening consequences; requiring urgent major care changes	Death

Salaets T, *et al. Arch Dis Child* 2019; 104:1167–1173. doi: 10.1136/archdischild-2019-317399

	(e.g. concomitant medication changed)	care changes (e.g. new medication or intervention)		
**Neonatal Sinus Bradycardia**<br>Definition C154933 | 10082188 (v22.0): *An abnormally low heart rate for age.*				
Brief, self-limiting, episodes of bradycardia; no care changes	Persistent bradycardia; no change in age-appropriate behavior; requiring minor care changes (e.g. concomitant medication adapted, intermittent increase FiO_2)	Persistent bradycardia; resulting in non-life threatening hemodynamic compromise; requiring major care changes (e.g. new medication or intervention)	Life-threatening consequences (e.g. shock); requiring urgent major care changes	Death
**Neonatal Tachyarrhythmia**<br>Definition C154948 | 10082055 (v22.0): *Non sinus rhythm with an abnormally high heart rate for age.*				
Brief, self-limiting, episodes of asymptomatic tachyarrhythmia (e.g. extrasystolic beats); no care changes	No change in age-appropriate behavior; requiring minor care changes (e.g. increased monitoring)	Resulting in non-life threatening hemodynamic compromise or changes in age-appropriate behavior; requiring major care changes (e.g. new medication or intervention)	Life-threatening consequences (e.g. shock); requiring urgent major care changes	Death
**Neonatal Bradyarrhythmia**<br>Definition C154949 | 10082054 (v22.0): *Non sinus rhythm with an abnormally low heart rate for age.*				
Brief, self-limiting, episodes of asymptomatic bradyarrhythmia; no care changes	No change in age-appropriate behavior; requiring minor care changes (e.g. increased monitoring)	Resulting in non-life threatening hemodynamic compromise; requiring major care changes (e.g. new medication or intervention)	Life-threatening consequences (e.g. shock); requiring urgent major care changes	Death
**Neonatal Edema**<br>Definition C154931 | 10054497: *Accumulation of an excessive amount of fluid in cells or intercellular tissues.*				
Mild edema; no change in age appropriate behavior; no care changes indicated	Moderate edema; no change in age appropriate behavior; requiring minor care changes (e.g. alteration in fluid management)	Severe edema; limiting age appropriate behavior; requiring major care changes (e.g. diuretics)	Life threatening consequences (e.g. respiratory failure, shock); requiring urgent major care changes (e.g. intubation, dialysis)	Death
**Neonatal Coagulation Disorder**<br>Definition C154921 | 10009732: *A condition of abnormal blood clotting or bleeding.*				
Minor biochemical coagulation abnormalities without clinical signs; no care changes indicated	Biochemical coagulation abnormalities with clinical signs; requiring increased monitoring	Biochemical or clinical coagulation abnormalities; requiring intervention	Life threatening consequences (e.g. severe pulmonary embolism, limb ischemia, hemorrhagic	Death

			shock, DIC); requiring urgent major care changes	

Respiratory

Grade 1	Grade 2	Grade 3	Grade 4	Grade 5
Mild	Moderate	Severe	Life threatening	Death
**Infantile Apnea**<br>Definition C154938 |10077322: *Cessation of air flow.*				
Self-limiting apnea	Apnea responsive to stimulation or intermittent FiO_2-increase.	Apnea requiring stimulation and sustained FiO_2 increase; requiring non-invasive ventilation; reoccurrences requiring start of or relevant increase in dose of respiratory stimulants or other major care changes	Life-threatening respiratory and/or hemodynamic compromise; (semi-)urgent intubation required	Death
**Neonatal Respiratory Insufficiency**<br>Definition C154951 | 10082057 (v22.0): *Significant impairment of gas exchange resulting in compensatory breathing efforts and eventually hypoxia and/or hypercarbia.**				
Clinical evidence of mildly increased respiratory distress (e.g. increased work of breathing) without significant deterioration in gas exchange (increase in pCO2 or decrease in oxygenation); no change in baseline functioning; no care changes required	Clinical evidence of increased respiratory distress without significant deterioration in gas exchange (increase in pCO2 or decrease in oxygenation); corrected by minor adjustments in current ventilatory support, supplemental oxygen or non-invasive support	Clinical evidence of increased respiratory distress with relevant deterioration in gas exchange (increase in pCO2 or decrease in oxygenation); requiring major care change (e.g. start invasive support)	Life-threatening respiratory and/or hemodynamic compromise; requiring urgent care change (e.g. urgent intubation)	Death
**Consider this adverse event for any patient whose respiratory condition deteriorates from baseline. If any specific diagnosis is identified: use diagnosis specific scale (e.g. RDS, pulmonary hemorrhage)*				
**Neonatal Respiratory Distress Syndrome** (RDS)<br>Definition C154950 | 10028974: *Progressive alveolar atelectasis from birth due to an abnormality of synthesis, function or metabolism of surfactant, characterized by respiratory failure and an abnormal chest radiograph showing diffuse reticulogranular densities and air bronchograms.*				
Radiological evidence without clinical signs; clinical evidence of mildly increased respiratory distress (e.g. increased work of breathing)	Clinical evidence of increased respiratory distress without significant deterioration in gas exchange (increase in pCO2 or decrease in	Clinical evidence of increased respiratory distress with relevant deterioration in gas exchange (increase in pCO2 or decrease in	Life-threatening respiratory and/or hemodynamic compromise; requiring urgent care change (e.g. urgent intubation)	Death

without significant deterioration in gas exchange (increase in pCO2 or decrease in oxygenation); no supportive care required	oxygenation); corrected by minor adjustments in current ventilatory support, supplemental oxygen or non-invasive support	oxygenation); requiring major care change (e.g. start invasive support)		
**Neonatal Pulmonary Hemorrhage**<br>Definition C154936 | 10082199 (v22.0): *Bleeding in the respiratory tract of a neonate.*				
Limited hemorrhagic secretion in ET tube	Hemorrhagic secretion in ET tube; without relevant increase in PCO_2 or decrease in oxygenation; requiring minor changes in care (e.g. increase PEEP)	Hemorrhagic secretion in ET tube; with relevant increase in PCO_2 or decrease in oxygenation; requiring major change in ventilatory support or transfusion	Life-threatening respiratory and/or hemodynamic compromise	Death
**Persistent Pulmonary Hypertension of the Newborn** (PPHN)<br>Definition C154940 | 10082195 (v22.0): *Elevated pulmonary vascular pressure in a neonate.*				
Technical evidence of increased RV-pressures; no clinical symptoms	Increased estimated RV-pressures; with moderate clinical symptoms; oxygenation index <25; minor care changes required	Increased estimated RV-pressures; with severe clinical symptoms; oxygenation index >25; major care changes required (e.g. iNO)	Life-threatening respiratory and/or hemodynamic compromise; ECMO required; oxygenation index >40	Death
**Neonatal Pneumothorax**<br>Definition C154920 | 10082056 (v22.0): *A collection of air or other gas between the visceral and parietal pleura.*				
Radiological evidence of pneumothorax; no clinical signs; no care change required	Radiological evidence of pneumothorax; minor clinical signs; minor care change required (e.g. oxygen, increased monitoring)	Radiological evidence of pneumothorax; with clinical signs; major care change required (e.g. chest drainage)	Life-threatening respiratory and/or hemodynamic compromise (e.g. tension pneumothorax); urgent major care change required	Death
**Bronchopulmonary Dysplasia**<br>Definition C154919 | 10006475: *A chronic lung disorder associated with pulmonary maldevelopment, scarring, and/or inflammation that develops in preterm neonates. The condition is defined based on treatment with supplemental oxygen for at least 28 days adjusted for the degree of prematurity.*				
Supplemental oxygen at 28 days AND Breathing room air at 36 weeks PMA in infants born <32 weeks' gestation; breathing room air by 56 days postnatal age in	Supplemental oxygen at 28 days AND Need for 22-30% oxygen at 36 weeks PMA in infants born <32 weeks' gestation; need for 22-30% oxygen by 56 days postnatal	Supplemental oxygen at 28 days AND Need for >30% oxygen or positive pressure at 36 weeks PMA in infants born <32 weeks' gestation; need for >30%	Supplemental oxygen at 28 days AND Need for >30% oxygen AND positive pressure at 36 weeks PMA in infants born <32 weeks' gestation; need for >30%	Death

infants born >32 weeks' gestation; breathing room air at discharge	age in infants born >32 weeks' gestation; need for 22-30% oxygen at discharge	oxygen or positive pressure by 56 days postnatal age in infants born >32 weeks' gestation; need for >30% oxygen and/or positive pressure at discharge	oxygen AND positive pressure by 56 days postnatal age in infants born >32 weeks' gestation; need for >30% oxygen AND positive pressure at discharge	
For conversion of oxygen administered by different modalities to FiO_2: see for example http://nicutools.org/MediCalcs/ActualO2.php3				

Gastro-intestinal

Grade 1	Grade 2	Grade 3	Grade 4	Grade 5
Mild	Moderate	Severe	Life threatening	Death
**Necrotizing Enterocolitis** (NEC)<br>Definition C154922 | 10052818: *A disease of neonates in which there is extensive mucosal ulceration, pseudomembrane formation, submucosal hemorrhage, and necrosis.*				
-	-	NEC confirmed; major care change indicated (e.g. NPO, antibiotics, non-urgent surgery)	Bowel perforation (pneumoperitoneum) (Bell IIIB); shock, DIC, combined respiratory and metabolic acidosis (Bell IIIA); urgent major care change indicated	Death
If NEC is not confirmed (Bell stages I): please record severity of individual symptoms (e.g. feeding intolerance).				
**Neonatal Diarrhea**<br>Definition C154953 | 10012731: *Watery bowel movements.*				
Increase of 2-4 stools per day over baseline; mild increase in ostomy output compared to baseline	Increase of 4 - 6 stools per day over baseline; moderate increase in ostomy output compared to baseline	Increase of >=7 stools per day over baseline; severe increase in ostomy output compared to baseline; signs of dehydration	Life-threatening consequences (e.g. severe dehydration)	Death
**Infantile Vomiting**<br>Definition C154942 | 10075315: *Expulsion of the contents of the stomach through the mouth.*				
Increase in vomiting over baseline, self-limiting.	Persistent increase in vomiting over baseline; no dehydration; minor changes in feeding support indicated	Persistent increase in vomiting over baseline; signs of dehydration; major changes in feeding support indicated (e.g. change to TPN or gavage)	Life-threatening consequences (e. g. severe dehydration)	Death
**Feeding Intolerance**<br>Definition C154944 | 10076042: *Inability to achieve a full feeding volume*.				
Mild feeding intolerance (e.g. increased gastric residual volume or	Moderate feeding intolerance; resulting in minor discomfort or	Severe feeding intolerance; requiring major changes in feeding	Life-threatening consequences	Death

abdominal distension); without discomfort; no change in care indicated	alteration of drinking behavior; minor care changes indicated (e.g. feeds withheld)	support indicated (e.g. change to TPN or gavage)		
**Neonatal Gastrointestinal Bleeding**<br>Definition C154943 | 10082192 (v22.0): *Hemorrhage originating at any site located within the gastrointestinal tract.*				
Mild, self-limiting, bleeding; no change in care required	Moderate bleeding; minor change in care or monitoring required	Severe bleeding; non-life threatening hemodynamic consequences; major care change required (e.g. invasive intervention, transfusion, long term medical treatment)	Life-threatening consequences (e.g.hemorrhagic shock); urgent major care change required	Death
**Neonatal Spontaneous Intestinal Perforation** (SIP)<br>Definition C154924 | 10082196: *A perforation in the gastrointestinal tract of a newborn with no demonstrable cause.*				
-	-	Presence of SIP, non-urgent medical stabilization and surgical intervention indicated	Life-threatening consequences (e.g. shock, organ failure); urgent intervention indicated	Death
**Neonatal Constipation**<br>Definition C154946 | 10082190 (v22.0): *Irregular and infrequent or difficult evacuation of the bowels.*				
Reduced number of stools or hard stools; no apparent discomfort	Reduced number of stools or hard stools; apparent discomfort; minor care changes indicated	Reduced number of stools or hard stools; signs of obstruction; apparent discomfort or affecting feeding; major care changes indicated (e.g. long term medical treatment, enema)	Life-threatening consequences	Death

Infectious

Grade 1	Grade 2	Grade 3	Grade 4	Grade 5
Mild	Moderate	Severe	Life threatening	Death
**Neonatal Culture Negative Sepsis**<br>Definition C154928 | 10082059 (v22.0): *A systemic inflammatory response without identifiable cause.*				
-	Suspected sepsis with mild or ambiguous signs;	Suspected sepsis with severe signs (e.g. fever, grunting); support	Life-threatening consequences (e.g. state of shock, DIC); urgent major	Death

	anti-infectives initiated	treatment escalated or initiated	care change required	
**Neonatal Culture Positive Sepsis**<br>Definition C154927 | 10082058 (v22.0): *A systemic inflammatory response to an infection.*				
Blood culture positive; no care change indicated (e.g. contamination suspected)	Blood culture positive with mild or ambiguous signs; anti-infectives initiated	Blood culture positive with severe signs; support treatment escalated or initiated	Life-threatening consequences (e. g. state of shock, DIC); urgent major care change required	Death

General

Grade 1	Grade 2	Grade 3	Grade 4	Grade 5
Mild	Moderate	Severe	Life threatening	Death
**Neonatal Rash**<br>Definition C154935 | 10037871: *An eruption in the skin which affects its appearance and/or texture.*				
Localized rash	Diffuse rash or target lesions	Diffuse rash and vesicles or limited number of bullae or superficial ulcerations of mucous membrane limited to one site	Extensive or generalized bullous lesions or ulceration of mucous membrane involving 2 or more distinct mucosal sites or Stevens-Johnson syndrome or toxic epidermal necrosis	Death
**Neonatal Administration Site Complication**<br>Definition C154947 | 10082193 (v22.0): *Local irritation or complication at the administration site of a drug.*				
Painless edema	Erythema with associated symptoms (e.g., edema, pain, induration, phlebitis)	Ulceration or necrosis; severe tissue damage; operative intervention indicated	Life-threatening consequences; urgent intervention indicated	Death
**Neonatal Fever**<br>Definition C154941 | 10016562: *Elevation of body temperature above normal due to the production of more heat than the body is able to dissipate.*				
Isolated, not sustained fever, no change in baseline age-appropriate behavior	Fever, minor change in baseline age-appropriate behavior; minor changes in care (e.g. environmental settings, symptomatic treatment)	Fever, major change in baseline age-appropriate behavior and without resolution to symptomatic treatment.	Life-threatening consequences	Death

Any other AE

Grade 1	Grade 2	Grade 3	Grade 4	Grade 5

Salaets T, *et al. Arch Dis Child* 2019; 104:1167–1173. doi: 10.1136/archdischild-2019-317399

Mild	Moderate	Severe	Life threatening	Death
**Any other AE**				
Mild; asymptomatic or mild symptoms; clinical or diagnostic observations only; no change in baseline age-appropriate behavior*; no change in baseline care or monitoring indicated	Moderate; resulting in minor changes of baseline age-appropriate behavior*; requiring minor changes in baseline care or monitoring***	Severe; resulting in major changes of baseline age-appropriate behavior* or non-life threatening changes in basal physiological processes**; requiring major change in baseline care or monitoring****	Life-threatening; Resulting in life-threatening changes in basal physiological processes**; requiring urgent major change in baseline care	Death related to AE
*Age-appropriate behavior refers to oral feeding behavior, voluntary movements and activity, crying pattern, social interactions and perception of pain.<br>**Basal physiological processes refer to oxygenation, ventilation, tissue perfusion, metabolic stability and organ functioning.<br>***Minor care changes constitute: brief, local, non-invasive or symptomatic treatments<br>***Major care changes constitute: surgery, addition of long term treatment, upscaling care level				
If the different factors of this scale result in conflicting severity grades, **the highest grade** should be reported.				

Salaets T, *et al. Arch Dis Child* 2019; 104:1167–1173. doi: 10.1136/archdischild-2019-317399
